# YAP in epithelium senses gut barrier loss to deploy defenses against pathogens

**DOI:** 10.1371/journal.ppat.1008766

**Published:** 2020-08-28

**Authors:** Yi-Cheng Ma, Zhong-Shan Yang, Lan-Qing Ma, Ran Shu, Cheng-Gang Zou, Ke-Qin Zhang

**Affiliations:** 1 Key Laboratory for Conservation and Utilization of Bio-Resources in Yunnan, School of Life Sciences, Yunnan University, Kunming, Yunnan, China; 2 Faculty of Basic Medicine, Yunnan University of Traditional Chinese Medicine, Kunming, Yunnan, China; 3 Yunnan Institute of Digestive Disease, Department of Digestive Diseases, The First Affiliated Hospital, Kunming Medical University, Kunming, Yunnan, China; 4 Department of Pathology, The First Affiliated Hospital, Kunming Medical University, Kunming, Yunnan, China; University of Massachusetts Medical School, UNITED STATES

## Abstract

Pathogens commonly disrupt the intestinal epithelial barrier; however, how the epithelial immune system senses the loss of intestinal barrier as a danger signal to activate self-defense is unclear. Through an unbiased approach in the model nematode *Caenorhabditis elegans*, we found that the EGL-44/TEAD transcription factor and its transcriptional activator YAP-1/YAP (Yes-associated protein) were activated when the intestinal barrier was disrupted by infections with the pathogenic bacterium *Pseudomonas aeruginosa* PA14. Gene Ontology enrichment analysis of the genes containing the TEAD-binding sites revealed that “innate immune response” and “defense response to Gram-negative bacterium” were two top significantly overrepresented terms. Genetic inactivation of *yap-1* and *egl-44* significantly reduced the survival rate and promoted bacterial accumulation in worms after bacterial infections. Furthermore, we found that disturbance of the E-cadherin-based adherens junction triggered the nuclear translocation and activation of YAP-1/YAP in the gut of worms. Although YAP is a major downstream effector of the Hippo signaling, our study revealed that the activation of YAP-1/YAP was independent of the Hippo pathway during disruption of intestinal barrier. After screening 10 serine/threonine phosphatases, we identified that PP2A phosphatase was involved in the activation of YAP-1/YAP after intestinal barrier loss induced by bacterial infections. Additionally, our study demonstrated that the function of YAP was evolutionarily conserved in mice. Our study highlights how the intestinal epithelium recognizes the loss of the epithelial barrier as a danger signal to deploy defenses against pathogens, uncovering an immune surveillance program in the intestinal epithelium.

## Author summary

The intestinal epithelial barrier is an important line of defense against pathogenic bacteria infecting the intestine. Persistent bacterial infections can cause disruption of the intestinal barrier; however, how the epithelia immune system recognizes the loss of intestinal barrier as a danger signal to activate self-defense against pathogens is unclear. Using the nematode *Caenorhabditis elegans* as a model animal, we show that the EGL-44/TEAD transcription factor and its transcriptional activator YAP-1/YAP (Yes-associated protein) are activated when the intestinal barrier is disrupted by bacterial infections. Gene Ontology enrichment reveals that EGL-44/TEAD orchestrates a complex host response composed of innate immune response and defense response to Gram-negative bacteria. Furthermore, our data demonstrate that YAP-1/YAP and EGL-44/TEAD are required for resistance to infections with pathogenic bacteria when the intestinal barrier is disrupted in worms and mice. Our study reveals a novel strategy for the intestinal epithelium to sense danger through its internal architecture and initiate innate immunity.

## Introduction

The intestinal epithelium forms a physical barrier to limit entry of pathogens, microbial antigens, and toxins from the luminal environment into the mucosal tissues [1–3]. The epithelial barrier function is regulated by an apical junctional complex, consisting of the apical tight junctions (TJs), the subjacent adherens junctions (AJs), and the underlying desmosomes [3, 4]. TJs are composed of multiple integral transmembrane proteins, such as claudins, occludins, junctional adhesion molecules, and tricellulin, and cytosolic scaffold proteins, such as cingulin and zonulae occludens (ZOs) [3]. AJs are composed of a cadherin (E-cadherin)-catenin complex and its associated proteins [4, 5]. The cytoplasmic tail of E-cadherin interacts with the Armadillo repeat protein β-catenin, which in turn binds α-catenin. TJs and AJs link epithelial cells to form an extremely effective barrier to macromolecules and bacteria [6].

In addition to their primary function as a physically protective barrier, the intestinal epithelial cells can deploy a variety of mechanisms to activate inflammatory and immune responses against microbial pathogens [6–9]. For example, secretory intestinal epithelial cells are capable of producing and secreting mucins and antimicrobial peptides [7, 9]. In addition, intestinal epithelia express various innate pattern recognition receptors (PRPs), such as Toll-like receptors (TLRs) and nucleotide-binding and oligomerization domain (NOD)-like receptors (NLRs), [6, 7, 9]. These receptors recognize a variety of microbial ligands or endogenous signals associated with pathogenesis, triggering transcriptional expression of inflammatory factors and antimicrobial peptides that coordinate the elimination of pathogens. Persistent bacterial infections cause disruption of the intestinal barrier [10–13]. Disruption of junction proteins occurs through degradation by bacterial proteases or by biochemical alterations, such as phosphorylation or dephosphorylation [14].

Accumulating evidence has demonstrated that in *Drosophila*, intestinal epithelial damage caused by toxins and bacterial infection induces proliferation and differentiation of intestine stem cells to replenish damaged cells [15, 16]. During epithelial cell damage, Yorkie is activated, and induces such proliferation of intestine stem cells [17, 18]. YAP (ortholog of Yorkie in *Drosophila*) is a major downstream effector of the Hippo signaling pathway [19–21]. The core components of Hippo-YAP signaling, initially discovered in *Drosophila*, are highly conserved in mammals [22]. During activation of this pathway, the MST1/2 (mammalian Ste20-like kinases 1/2, orthologs of Hippo in *Drosophila*) activates the downstream kinase LATS1/2 (large tumor suppressor 1/2, orthologs of Warts in *Drosophila*). LATS1/2 then phosphorylates YAP, resulting in its cytoplasmic retention. If Hippo signaling is reduced, the dephosphorylated YAP is translocated into the nucleus, where it binds to and activates the transcription factor TEAD (TEA Domain). Accumulating evidence indicates that YAP interacts with some of the components of the apical junctional complex, such as angiomotin (AMOT) family proteins [23, 24] and α-catenin [25, 26]. Together, these proteins in TJs and AJs modulate cell proliferation through regulating YAP activity [23–26].

Recent studies have demonstrated that YAP is involved in innate immune responses to a variety of pathogens. For instance, verteporfin, an inhibitor of YAP/TEAD interactions, renders mice susceptible to gut infection by the helminth parasite *Trichuris muris*, suggesting a protective role of the YAP-TEAD complex in epithelial cells [27]. In contrast, flies with overexpression of Yorkie in fat bodies exhibit susceptibility to infection by Gram-positive bacteria [28]. In the fat body of flies, Yorkie mediates expression of the *Drosophila* IkB homolog, Cactus, which in turn suppresses the NF-kB family transcription factors Dorsal (Dl) and Dorsal-related immune factor (Dif) by retaining the latter in the cytoplasm. Likewise, an inhibitory role of YAP in the NF-kB signaling has also been demonstrated in mammals [29, 30]. In addition, YAP has been identified as a negative regulator of host antiviral responses in mammalian cells [31, 32]. YAP prevents the phosphorylation and nuclear translocation of the transcription factor IRF3, thus inhibiting IRF3-mediated production of type I interferon and interferon-stimulated genes in response to virus infection.

The model organism *Caenorhabditis elegans* is capable of detecting infection and protecting itself from diverse pathogens [33–35]. In this report, we took advantage of the simple intestine of *C*. *elegans* to elucidate the role of the intestinal epithelium in host defense against bacterial infection when the epithelial barrier is disrupted. Here we reported that the YAP-TEAD complex was crucial for host defense against bacterial infections. YAP-1/YAP was activated when the E-cadherin complex was disturbed and the intestinal barrier was disrupted during bacterial infections. Mutations in *egl-44* and *yap-1* led to a defect in host defense against pathogen infections in *C*. *elegans*. Similarly, we showed that YAP was also activated in the gut of mice during loss of the intestinal barrier integrity and required for resistance to bacterial infections. Thus, EGL-44/TEAD and YAP-1/YAP play an important role in innate immune responses to bacterial infections.

## Results

### EGL-44/TEAD transcription factor is involved in host response to disruption of barrier during infection

**Fig 1:**
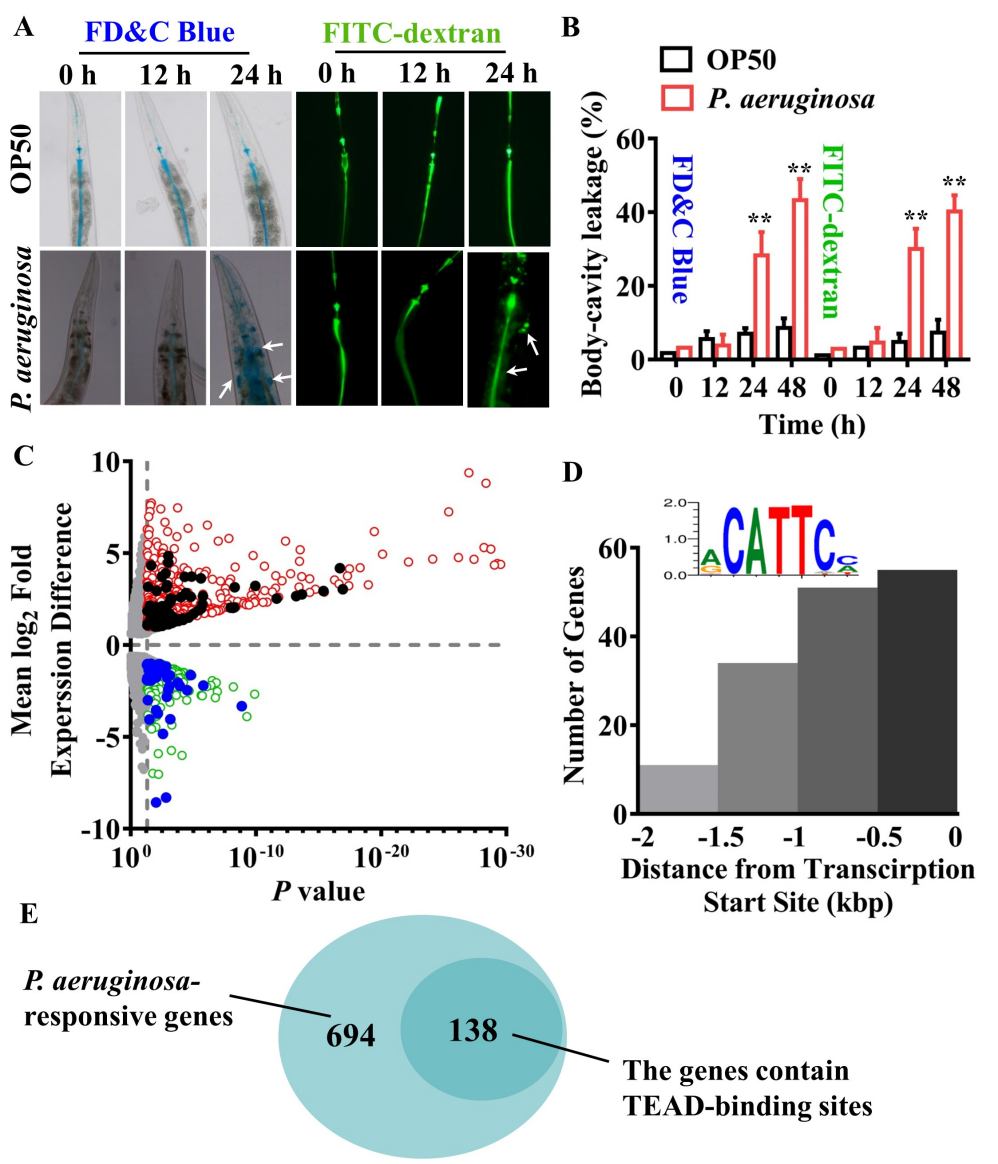
EGL-44/TEAD transcription factor responses to disruption of intestinal barrier induced by *P*. *aeruginosa*. **(A)** Intestinal permeability measured by food dye FD&C Blue No. 1 (FD&C Blue) and FITC-dextran staining in wild type (WT) worms exposed to *P*. *aeruginosa* PA14 or fed *E*. *coli* OP50 at 0 h, 12 h, and 24 h, respectively. **(B)** Quantification of body-cavity leakages was measured in animals over time. These results are means ± SD of three independent experiments (n ≥ 50 worms per experiment). **P < 0.01 relative to 0 h (One-way ANOVA followed by a Student-Newman-Keuls test). **(C)** Volcano Plot for differential gene expression in worms exposed to *P*. *aeruginosa*. The red dots represent the significantly upregulated genes and the green dots represent the significantly downregulated genes. The black and blue dots represent the 92 upregulated genes and 46 downregulated genes whose promoters contain the TEAD-binding sites, respectively. **(D)** Distribution of the identified CATTCC DNA motif in the promoter regions of genes in *C*. *elegans* responsive to *P*. *aeruginosa* infection. Insert shows logo representation of identified CATTCC DNA motif. **(E)** A Venn diagram comparing the overlap in *P*. *aeruginosa*-regulated genes and the genes whose promoters contain the TEAD-binding sites.

We first used two dyes, a food dye FD&C Blue No. 1 and the fluorescein isothiocyanate (FITC)-dextran of 40 kD, to evaluate intestinal epithelial barrier disruption [36]. A significant increase in permeability of the barrier was detected in worms at 24 and 48 hours, but not 12 hours, after infection with the Gram-negative bacterium *P*. *aeruginosa* PA14 (Fig 1A and 1B). In contrast, the barrier integrity remained intact in worms fed the standard laboratory food bacterium *Escherichia coli* OP50 (Fig 1A and 1B). Similar results were obtained from worms infected with another Gram-negative bacterium, *Salmonella enterica* serovar Typhimurium 468 (S1A and S1B Fig).

Two previous studies using whole-genome microarrays revealed that a set of immune response genes, such as C-type lectins, lysozymes, and CUB-like genes, were significantly induced during *P*. *aeruginosa* PA14 infection [37, 38]. To clarify the mechanisms of innate immune responses mediated by epithelial barrier disruption, we used RNA-Seq analysis to study the transcriptional profiles of worms at 24 hours after *P*. *aeruginosa* PA14 infection versus worms fed *E*. *coli* OP50. Among the 832 differentially expressed genes, 593 were upregulated, and 239 were downregulated in wild type (WT) animals (*P* < 0.05 and fold change cut-off > 2.0) (Fig 1C; S1 and S2 Tables). These results revealed the host’s broad transcriptional response to *P*. *aeruginosa* infection during disruption of the intestinal barrier. To identify transcription factors for host-response induction during intestinal barrier disruption, we investigated which transcription factor binding sites were overrepresented in the promoters of these 832 genes using the BioProspector program [39]. After analyzing 2 kb upstream of the transcription start sites (TSS), we identified an overrepresentation of the CATTCC DNA motif (*P* <1.58E-05) (Fig 1D; S3 Table). Of these *P*. *aeruginosa*-regulated genes, the promoters of 138 genes contain the TEAD-binding sites (Fig 1E; S3 Table). The CATTCC motifs were preferentially localized within the first 1000 bp upstream of potential target TSS (Fig 1D). This DNA sequence is a consensus motif recognized by TEAD transcription factors, the downstream effectors of the Hippo signaling [19–21]. In the *C*. *elegans* genome, the gene *egl-44* encodes the TEAD homolog [40]. We compared these 138 differentially expressed genes containing the TEAD-binding site with *P*. *aeruginosa* PA14 transcriptional responses at 4 hours from a previous study [41]. Of these 138 genes, 36 genes were upregulated and 19 genes downregulated at both 4 hours and 24 hours, whereas 56 genes were upregulated and 27 genes downregulated only at 24 hours, but unchanged at 4 hours (S2 Fig). Therefore, the 138 genes were classified as two clusters: the genes of Cluster I are regulated probably by other transcription factors at 4 hours and by the TEAD/EGL-44-YAP-1/YAP complex at 24 hours, while the genes of Cluster II are regulated mainly by the TEAD/EGL-44-YAP-1/YAP complex at 24 hours during *P*. *aeruginosa* PA14 infection. These results suggest that the TEAD/EGL-44-YAP-1/YAP complex is important for host-response induction during disruption of intestinal barrier.

### EGL-44/TEAD and YAP-1/ YAP regulates immune response-related genes

**Fig 2:**
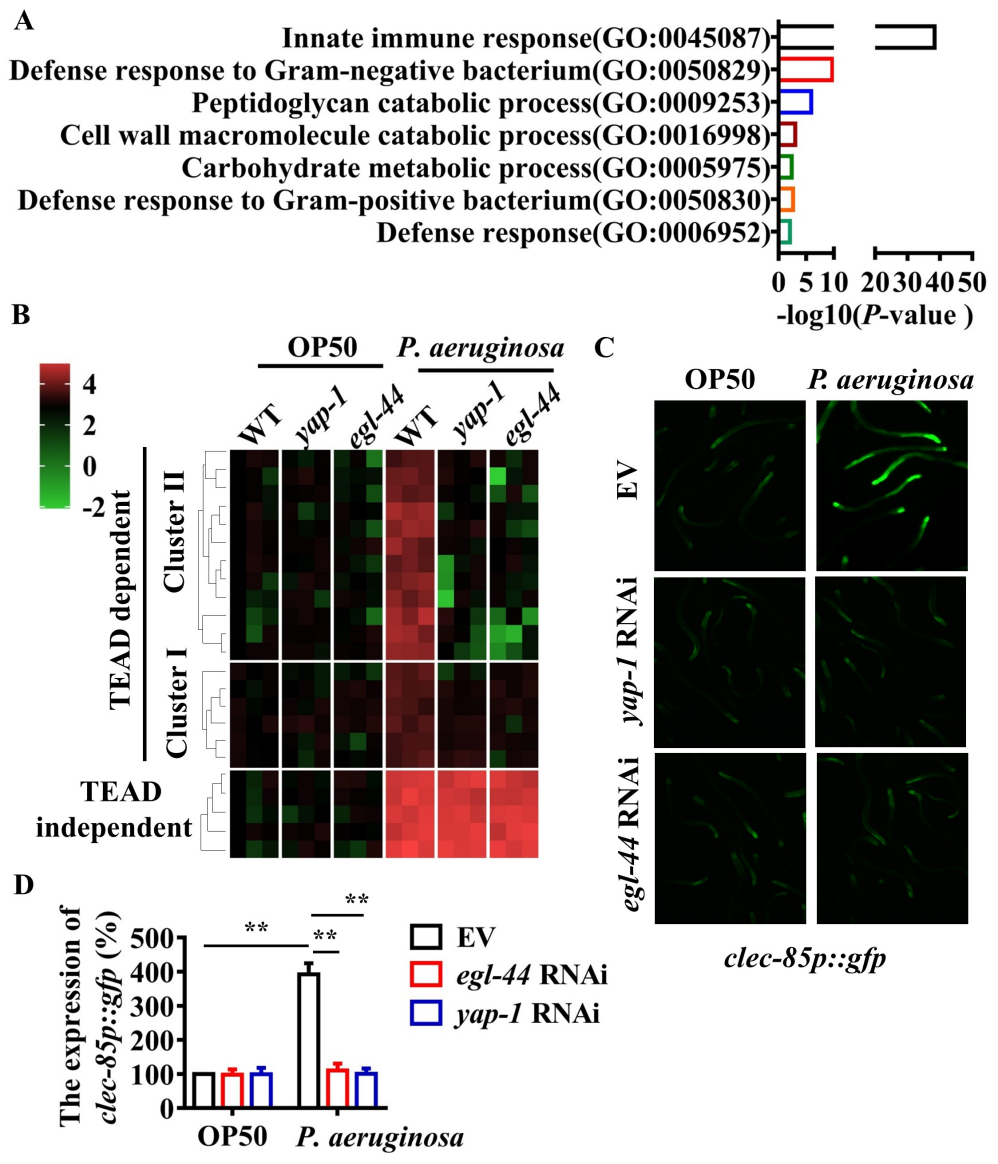
EGL-44/TEAD and YAP-1/YAP regulate the expression of immune-related genes after *P*. *aeruginosa* infection. **(A)** Enrichment analysis of the GO biological process of the *P*. *aeruginosa*-regulated genes containing CATTCC DNA motifs is shown. The enrichment *P* value of each term was transformed to a -log_10_^(*P*-value)^. **(B)** Unsupervised hierarchical clustering of expression levels (qRT-PCR) of YAP-1-EGL-44 complex-dependent immune-related genes as well as YAP-1-EGL-44 complex-independent genes in worms exposed to *P*. *aeruginosa* (24 h) by using Origin 2019b. Each column represents an independent replicate. **(C)** Expression of *clec-85p*::*gfp* was upregulated in worms infected with *P*. *aeruginosa* compared to worms fed *E*. *coli* OP50. Knockdown of *egl-44* and *yap-1* by RNAi reduced such an increase of *clec-85p*::*gfp* expression. **(D)** Quantification of GFP levels. These results are means ± SD of three independent experiments. **P < 0.01 relative to OP50 (n ≥ 50 worms per experiment) (One-way ANOVA followed by a Student-Newman-Keuls test).

Gene Ontology enrichment of the 138 genes containing the TEAD-binding via DAVID revealed that “Innate immune response” (GO: 0045087, *P* < 3.8E-39) and “Defense response to Gram-negative bacterium” (GO: 0050829, P < 2.5E-19) were two top significantly overrepresented terms (Fig 2A). Of these 39 “innate immune response” genes, 35 genes were upregulated and 4 genes were downregulated after *P*. *aeruginosa* PA14 infection. Of these 39 genes in the GO term category “Innate immune response”, 26 and 13 belonged to Clusters I and II, respectively. Using qRT-PCR, we tested the expression of 6 upregulated genes of Cluster I (*clec-67*, *clec-85*, *tsp-1*, *C49C8*.*5*, *T25D10*.*1*, and *Y46D2A*.*2*) and 12 upregulated genes of Cluster II (*clec-83*, *clec-86*, *fbxa-30*, *fbxa-60*, *prx-11*, *zip-10*, *F35E12*.*2*, *F39G3*.*5*, *F54E2*.*1*, *M04C3*.*2*, *valv-1*, and *clec-264*) 24 h after *P*. *aeruginosa* PA14 infection. We found that the mRNA levels of these immune response-related genes were significantly increased in WT worms exposed to *P*. *aeruginosa* PA14 for 24 hours, compared with those in worms fed *E*. *coli* OP50 (Fig 2B). However, the expression of these genes was downregulated by mutations in *yap-1(tm1416)* and *egl-44(mt2247)* mutants after *P*. *aeruginosa* PA14 infection. In contrast, mutations in *yap-1(tm1416)* and *egl-44(mt2247)* did not influence their expression in worms fed *E*. *coli* OP50 (Fig 2B). Meanwhile, we found that knockdown of *yap-1* and *egl-44* reduced the expression of *clec-85p*::*gfp* after *P*. *aeruginosa* PA14 infection (Fig 2C and 2D). Finally, we randomly selected 5 upregulated genes without a TEAD binding site (*dod-21*, *pgp-7*, *F35E12*.*5*, *clec-45*, and *Y51B9A*.*8*) and tested their expression after *P*. *aeruginosa* PA14 infection. We found that expression of these five genes was upregulated, which was independent of the EGL-44/TEAD-YAP-1/YAP complex (Fig 2B). These results demonstrate that the EGL-44/TEAD-YAP-1/YAP complex specifically controls a set of immune response-related genes in *C*. *elegans*.

### Infection induces nuclear accumulation of YAP-1/YAP in worms during disruption of intestinal epithelial barrier

**Fig 3:**
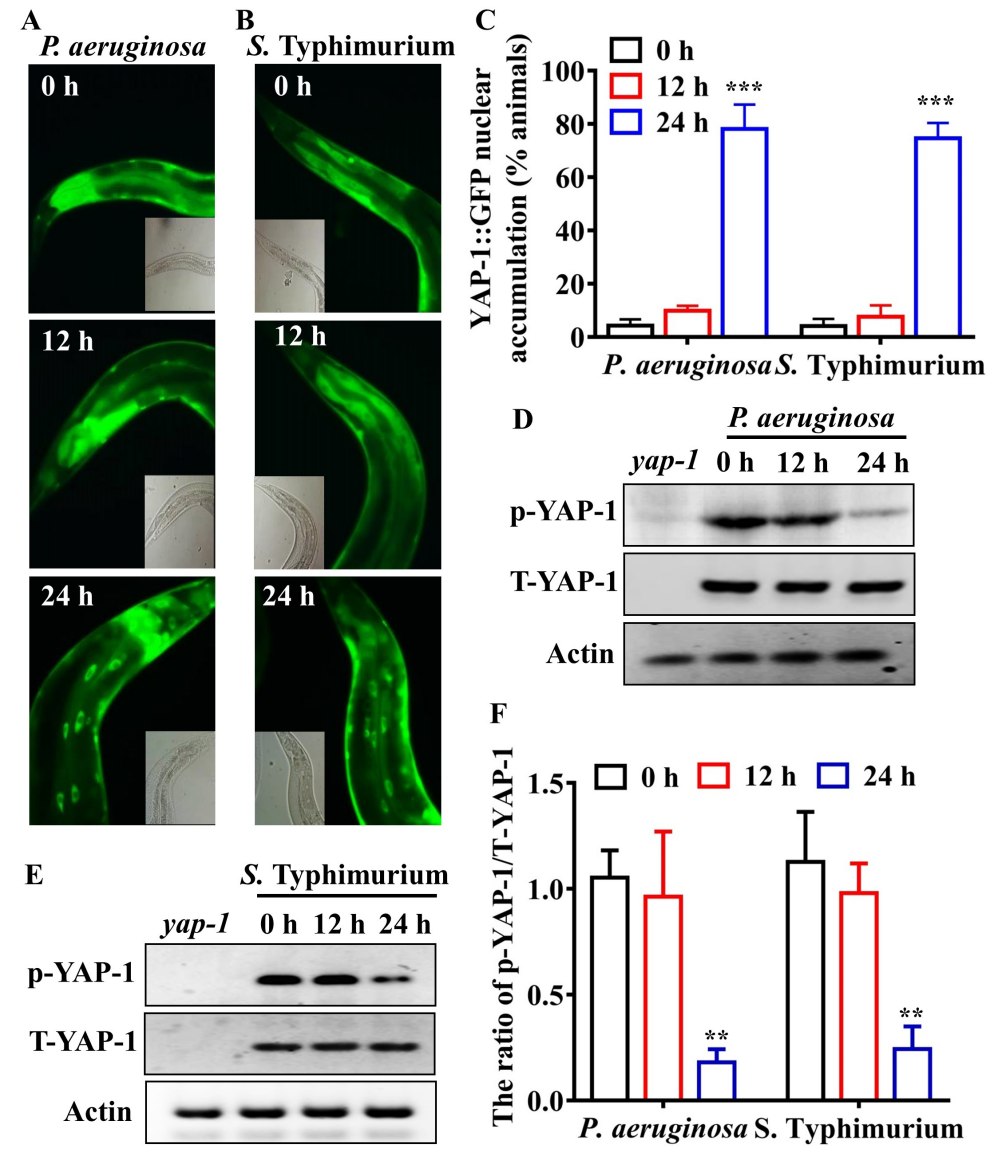
YAP-1/YAP is activated during disruption of intestinal barrier induced by pathogens. **(A and B)** *P*. *aeruginosa* **(A)** or *S*. Typhimurium **(B)** infection led to nuclear translocation of YAP-1::GFP in the intestine of worms. **(C)** Quantification of YAP-1 nuclear accumulation (n = 3 independent experiments, n = 100/condition). ***P < 0.001 relative to 0 h (One-way ANOVA followed by a Student-Newman-Keuls test). **(D and E)** The levels of phosphorylated YAP-1 were reduced in wild type (WT) worms after *P*. *aeruginosa* **(D)** or *S*. Typhimurium **(E)** infection. The blot is typical of three independent experiments. *yap-1*, *yap-1(tm1416)* mutants. **(F)** Quantification of the ratio of p-YAP-1 to total YAP-1. These results are means ± SD of three experiments. **P < 0.01 relative to 0 h (One-way ANOVA followed by a Student-Newman-Keuls test).

The transcriptional activity of TEAD requires YAP as a transcriptional coactivator [42, 43]. Thus, the upregulation of the TEAD/EGL-44-mediated immune response is probably due to activation of YAP-1, the *C*. *elegans* orthologue of YAP [40]. To test this hypothesis, we examined the effect of pathogen infections on the nuclear translocation of YAP-1/YAP. Using the transgenic worms expressing *yap-1p*::*yap-1*::*gfp*, we observed that, under standard growth conditions, YAP-1/YAP was mainly located in the cytoplasm in all tissues. However, infections by *P*. *aeruginosa* PA14 and *S*. Typhimurium all induced significant nuclear translocation of YAP-1/YAP in the intestine at 24 hours but not 12 hours after infection (Fig 3A–3C). Western blotting analysis revealed that the levels of phosphorylated YAP-1/YAP were markedly reduced at 24 hours but not 12 hours after infections by *P*. *aeruginosa* PA14 (Fig 3D and 3F) and *S*. Typhimurium (Fig 3E and 3F). These results demonstrate that pathogen infections activate YAP-1/YAP in *C*. *elegans* during disruption of intestinal barrier.

### EGL-44/TEAD and YAP-1/YAP are required for resistance to pathogens

**Fig 4:**
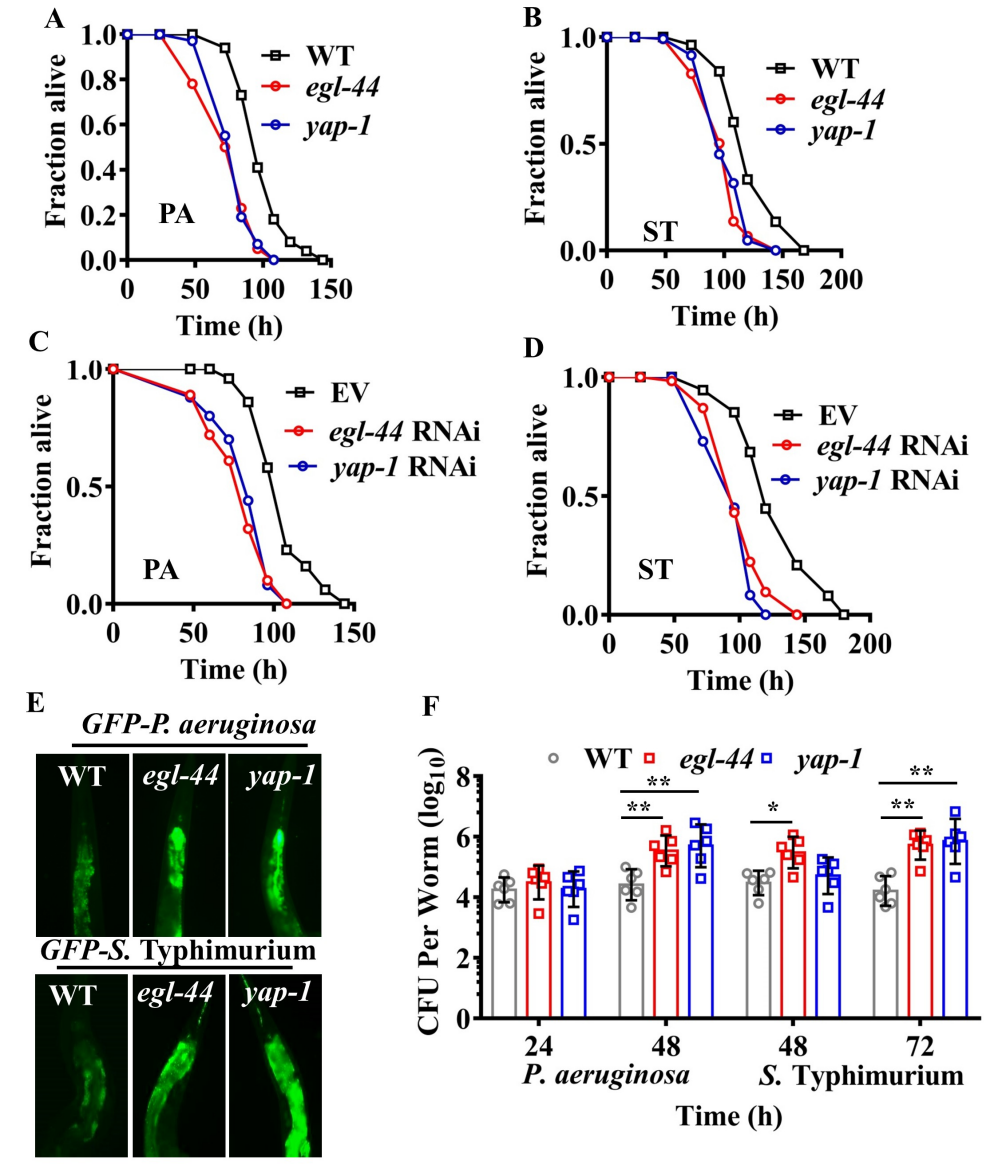
EGL-44/TEAD and YAP-1/YAP are required for host defense against pathogen infections. **(A and B)** *egl-44* and *yap-1* mutants were more susceptible to killing by *P*. *aeruginosa* (PA) **(A)** or *S*. Typhimurium (ST) **(B)** than wild type worms (WT). *P* < 0.01 relative to WT (Log-rank test). **(C and D)** Knockdown of *yap-1* and *egl-44* led to enhanced susceptibility to *P*. *aeruginosa* **(C)** or *S*. Typhimurium **(D)** infection. *P* < 0.01 relative to empty vector (EV) (Log-rank test). **(E and F)** Mutations in *egl-44* and *yap-1* promoted accumulation of pathogens. Fluorescence of worms exposed to *P*. *aeruginosa* for 48 h or *S*. Typhimurium expressing GFP for 72 h is shown **(E)**. The images are representative of three independent experiments. Colony-forming units (CFU) of *P*. *aeruginosa* and *S*. Typhimurium were measured in worms **(F)**. These results are mean ± SD of six independent experiments (n ≥ 50 worms per experiment). *P < 0.05, **P < 0.01 relative to WT (Two-sample t-test).

We next addressed the question of whether YAP-1/YAP and EGL-44/TEAD were involved in resistance to pathogenic bacteria. We found that *egl-44(mt2247)* and *yap-1(tm1416)* mutants were more sensitive to killing by *P*. *aeruginosa* PA14 (Fig 4A) and *S*. Typhimurium (Fig 4B) than WT worms. Likewise, *egl-44* and *yap-1* RNAi recapitulated the mutant phenotype (Fig 4C and 4D). To determine whether the susceptibility was attributable to accelerated bacterial infections in *yap-1* and *egl-44* mutants, we tested bacterial accumulation in the intestine, and found that the accumulation of *P*. *aeruginosa* PA14 or *S*. Typhimurium expressing GFP in *yap-1(tm1416)* and *egl-44(mt2247)* worms was significantly higher than that in WT worms (Fig 4E). Furthermore, mutations in *yap-1(tm1416)* and *egl-44(mt2247)* markedly increased the colony forming units (CFU) of *P*. *aeruginosa* or *S*. Typhimurium in worms (Fig 4F). Finally, we found that the worms overexpressing *yap-1p*::*yap-1*::*gfp* were more resistant to infections with *P*. *aeruginosa* PA14 (S3A Fig) and *S*. Typhimurium (S3B Fig) than WT worms. In addition, CFU of *P*. *aeruginosa* PA14 or *S*. Typhimurium in the worms overexpressing *yap-1p*::*yap-1*::*gfp* were significantly lower than those in WT worms (S3C Fig). These results suggest that YAP-1/YAP and EGL-44/TEAD are required for defense against bacterial pathogens. Previously, Iwasa et al. reported that a mutation in *yap-1(tm1416)* extended the lifespan of worms, while *yap-1* overexpression shortened the lifespan [40]. Thus, these results indicate that lifespan is not a major factor to determine pathogen susceptibility in *yap-1(tm1416)* mutants.

### Disturbance of the E-cadherin-catenin complex leads to activation of YAP-1/YAP

**Fig 5:**
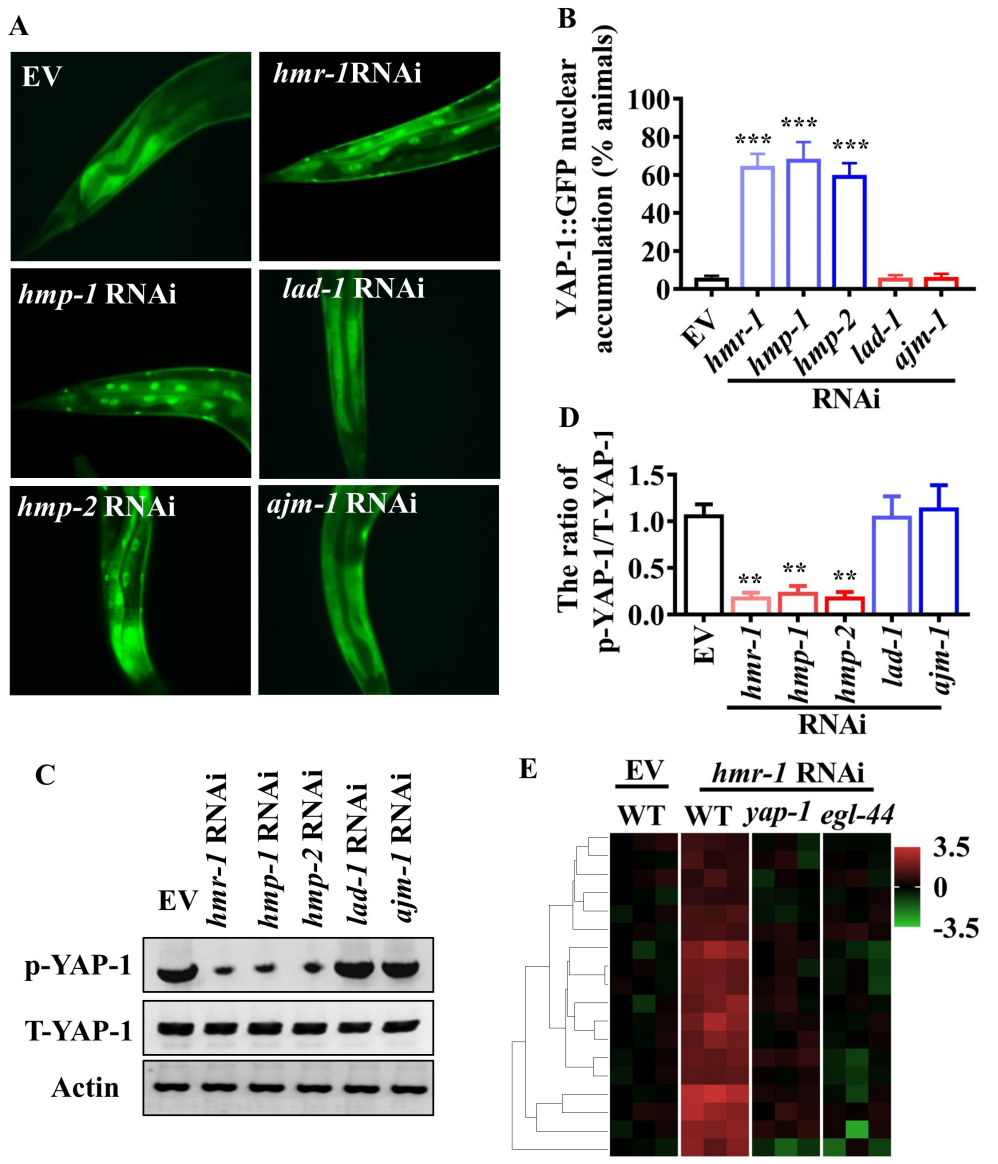
Disruption of adherens junction components leads to activation of YAP-1/YAP. **(A)** Knockdown of *hmr-1*, *hmp-1*, and *hmp-2*, but not *lad-1* and *ajm-1* by RNAi induced nuclear translocation of YAP-1::GFP in worms fed nonpathogenic *E*. *coli* OP50. **(B)** Quantification of YAP-1 nuclear accumulation (n = 3 independent experiments, n = 100/condition). ***P < 0.001 relative to empty vector (EV) (Two-sample t-test). **(C)** The levels of phosphorylated YAP-1 (p-YAP-1). The blot is typical of three independent experiments. **(D)** Quantification of the ratio of p-YAP-1 to total YAP-1 (T-YAP-1). These results are means ± SD of three experiments. **P < 0.01 relative to EV (Two-sample t-test). **(E)** Unsupervised hierarchical clustering of expression levels (qRT-PCR) of immune-related genes in *hmr-1* RNAi-treated worms fed *E*. *coli* OP50 (24 h) by using Origin 2019b. Each column represents an independent replicate. Mutations in *yap-1(tm1416)* and *egl-44(mt2247)* reduced the expression of these genes in the worms.

Accumulating evidence indicates that the apical junctional complex modulates epidermal cells or epithelial proliferation through regulating YAP activity [23–26]. A strong temporal relationship between the permeability of intestinal barrier and the nuclear translocation of YAP-1/YAP raises the possibility that disruption of the epithelial barrier by bacterial infection could activate YAP-1/YAP. To test this hypothesis, we used RNAi to silence the major genes encoding TJ proteins (LAD-1/LiCAM and AJM-1), and the genes encoding AJ proteins (HMR-1/E-cadherin, HMP-1/α-catenin, and HMP-2/β-catenin) [44], in worms fed *E*. *coli* OP50. We found that genetic inactivation of *hmr-1*, *hmp-1*, and *hmp-2*, but not *lad-1* and *ajm-1*, significantly enhanced the nuclear translocation of YAP-1/YAP (Fig 5A and 5B), and reduced the levels of phosphorylated YAP-1/YAP in worms fed *E*. *coli* OP50 (Fig 5C and 5D). To further confirm the activation of YAP-1/YAP, we determined the expression of the 6 Cluster I genes and 12 Cluster II genes mentioned above in worms subjected to *hmr-1* RNAi under normal growth conditions. We found that knockdown of *hmr-1* upregulated their expression (Fig 5E). However, mutations in *yap-1(tm1416)* and *egl-44(mt2247)* inhibited the upregulation of these genes in worms. Likewise, knockdown of *yap-1* and *egl-44* by RNAi reduced the expression of *clec-85p*::*gfp* in worms treated with *hmr-1* RNAi under normal growth conditions (S4 Fig). Next, using transgenic worms expressing *hmr-1p*::*hmr-1*::*gfp* or *hmp-1p*::*hmp-1*::*gfp*, we addressed the question of whether bacterial infections can influence the distribution of HMR-1 and HMP-1 of AJ proteins. HMR-1::GFP and HMP-1::GFP distribution showed the rectangular form in the intestine without bacterial infections (S5A Fig). Disturbance of HMR-1::GFP and HMP-1::GFP distribution became pronounced at 24 hours, but not 12 hours, after *P*. *aeruginosa* PA14 infection (S5A Fig). Similar results were obtained in worms exposed to *S*. Typhimurium (S5B Fig). These results suggest that disruption of the E-cadherin-β-catenin-α-catenin-YAP complex leads to the activation of YAP-1/YAP after bacterial infections.

### PP2A is crucial for the activation of YAP-1/YAP during infection-induced barrier loss

Activation of the Hippo pathway induces the phosphorylation of YAP by the serine/threonine kinase LATS1/2, leading to cytoplasmic sequestration of YAP [22, 42]. We hypothesized that *P*. *aeruginosa* could promote nuclear translocation of YAP by inactivating the Hippo signaling. However, we found that the phosphorylation levels of WTS-1, the *C*. *elegans* orthologue of LATS1/2, were not altered after *P*. *aeruginosa* PA14 infection (S6A Fig), suggesting that there was no significant change in the activation status of WTS-1/LATS1/2. Meanwhile, although knockdown of *hmr-1*, *hmp-1*, and *hmp-2* activated YAP-1/YAP in worms fed *E*. *coli* OP50 (Fig 5A–5C), it failed to influence the phosphorylation levels of WTS-1/LATS1/2 (S6B Fig). These results suggested that the activation of YAP-1/YAP might be independent of the Hippo pathway during disruption of intestinal barrier.

**Fig 6:**
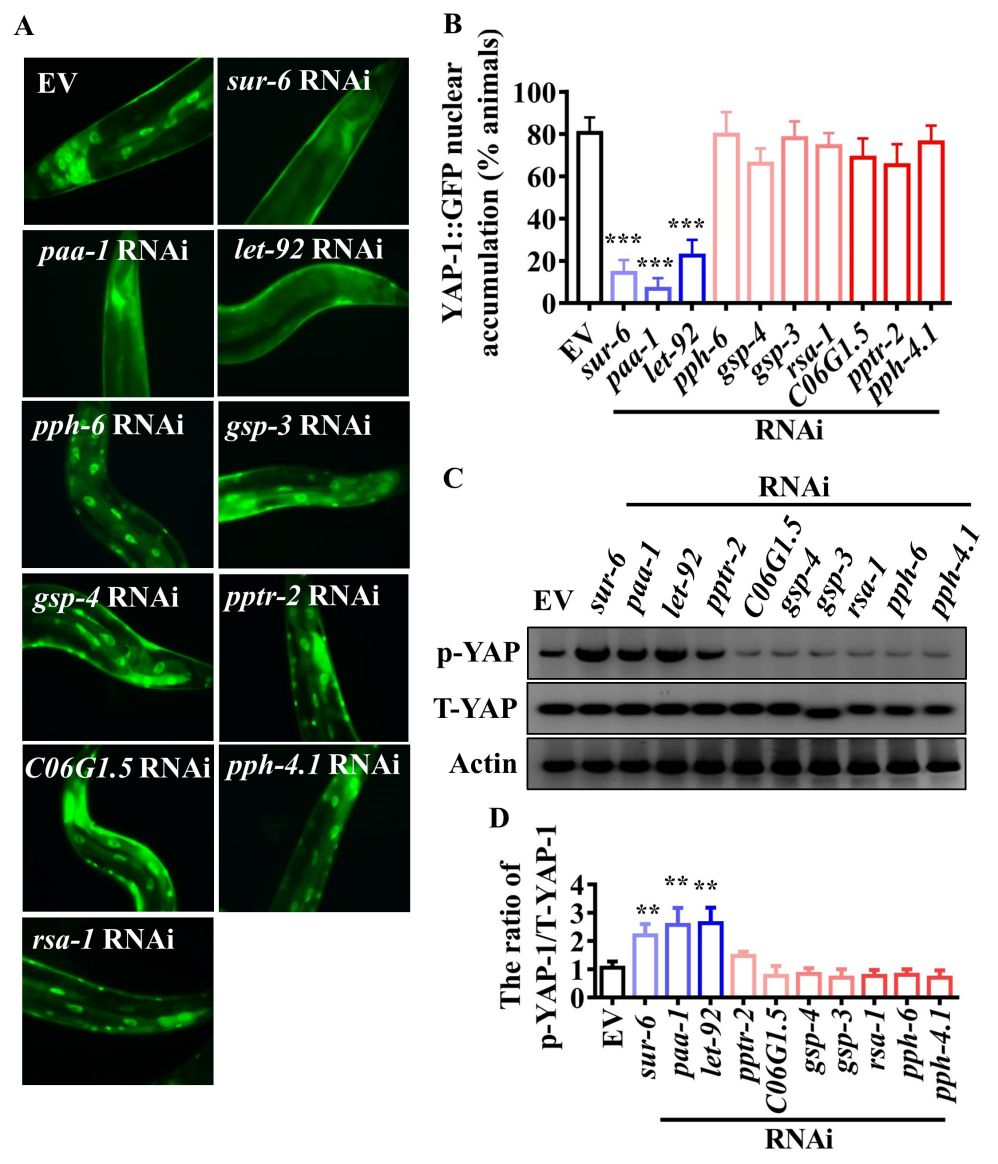
PP2A is involved in activation of YAP-1/YAP in worms during *P*. *aeruginosa* infection. **(A)** Knockdown of *let-92*, *paa-1*, and *sur-6*, but not *pptr-2*, *ras-1*, *C06G1*.*5*, *gsp-3*, *gsp-4*, *pph-4*.*1*, and *pph-6*, by RNAi suppressed the nuclear translocation of YAP-1 at 24 h after *P*. *aeruginosa* infection. **(B)** Quantification of YAP-1 nuclear accumulation (n = 3 independent experiments, n = 100/condition). ***P < 0.001 relative to empty vector (EV) (Two-sample t-test). **(C)** The levels of phosphorylated YAP-1 (p-YAP-1) detected by western blotting. The blot is typical of three independent experiments. **(D)** Quantification of the ratio of p-YAP-1 to total YAP-1 (T-YAP-1). These results are means ± SD of three experiments. **P < 0.01 relative to EV (Two-sample t-test).

In murine epidermal cells, loss of α-catenin leads to an efficient association of YAP and PP2Ac, the catalytic subunit of the protein phosphatase 2A (PP2A) [25]. PP2Ac mediates the dephosphorylation of YAP, thereby triggering its activation. We thus tested the role of serine/threonine phosphatases in the activation of YAP-1/YAP. We used RNAi to silence serine/threonine phosphatase genes including *gsp-3* and *gsp-4* (the *C*. *elegans* homologs of PP1), *let-92* (the *C*. *elegans* homolog of PP2Ac, the catalytic subunit of PP2A), *paa-1* (the *C*. *elegans* homolog of PP2Aa, the structural subunit of PP2A), *sur-6*, *pptr-2*, *ras-1* and *C06G1*.*5* (the *C*. *elegans* homologs of PP2Ab, the structural subunits of PP2A), *pph-4*.*1* (the *C*. *elegans* homolog of PP4), and *pph-6* (the *C*. *elegans* homolog of PP6). We found that the knockdowns of *let-92*, *paa-1*, and *sur-6*, but not *pptr-2*, *ras-1*, *C06G1*.*5*, *gsp-3*, *gsp-4*, *pph-4*.*1*, and *pph-6*, significantly suppressed the nuclear translocation of YAP-1/YAP at 24 hours after *P*. *aeruginosa* infection (Fig 6A and 6B). Meanwhile, knockdowns of *let-92*, *paa-1*, and *sur-6*, but not other phosphatase genes, substantially restored the phosphorylation levels of YAP-1/YAP at 24 hours after *P*. *aeruginosa* infection (Fig 6C and 6D). Next, we tested whether the expression of the 12 Cluster II immune response genes mentioned above was dependent on *let-92*, *paa-1*, or *sur-6*. We found that the mRNA levels of these genes were downregulated in worms subjected to *let-92*, *paa-1*, or *sur-6* RNAi after *P*. *aeruginosa* PA14 infection (S7A Fig). Meanwhile, knockdown of *let-92*, *paa-1*, or *sur-6* RNAi markedly accelerated worm death and increased CFU of *P*. *aeruginosa* PA14 in worms (S7B and S7C Fig). These results suggest that PP2A is involved in the activation of YAP-1/YAP and required for defense against *P*. *aeruginosa* infection.

**Fig 7:**
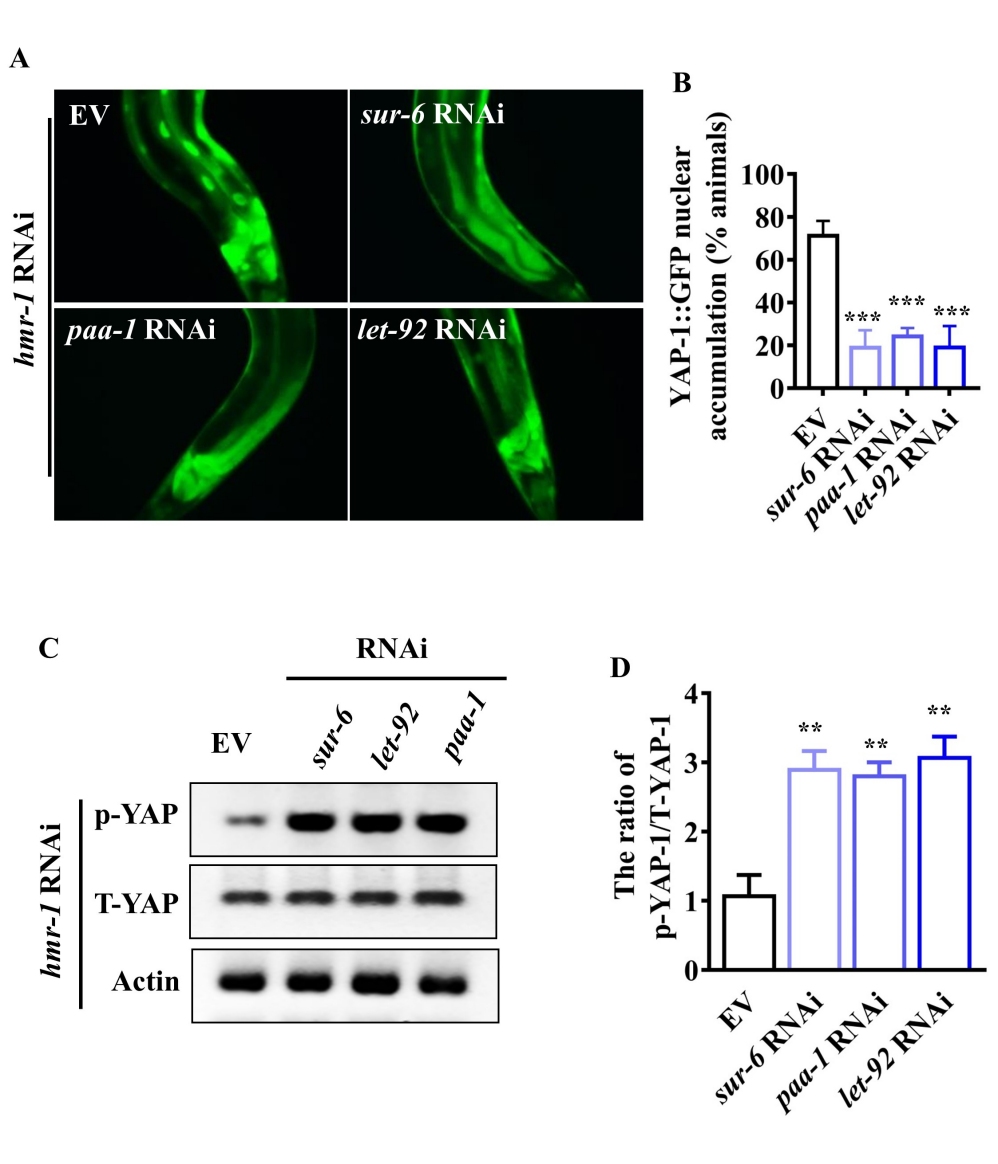
PP2A is required for activation of YAP-1/YAP after knockdown of *hmr-1* fed *E*. *coli* OP50. **(A)** Knockdown of *let-92*, *paa-1*, and *sur-6* suppressed the nuclear translocation of YAP-1/YAP in worms subjected to *hmr-1* RNAi. **(B)** Quantification of YAP-1 nuclear accumulation (n = 3 independent experiments, n = 100/condition). ***P < 0.001 relative to empty vector (EV) (Two-sample t-test). **(C)** Knockdown of *let-92*, *paa-1*, and *sur-6* restored the phosphorylation of YAP-1 in *hmr-1* RNAi worms. The blot is typical of three independent experiments. **(D)** Quantification of the ratio of phosphorylated YAP-1 (p-YAP-1) to total YAP-1 (T-YAP-1). These results are means ± SD of three experiments. **P < 0.01 relative to EV (Two-sample t-test).

Next, we tested the role of PP2A in the activation of YAP-1/YAP in worms subjected to genetic inactivation of *hmr-1* under normal growth conditions. Knockdowns of *let-92*, *paa-1*, and *sur-6* by RNAi markedly inhibited the nuclear translocation of YAP-1/YAP (Fig 7A and 7B). Again, knockdowns of *let-92*, *paa-1*, and *sur-6* substantially restored the phosphorylation levels of YAP-1/YAP in *hmr-1* RNAi worms (Fig 7C and 7D). These results suggest that PP2A is required for the activation of YAP-1/YAP during disruption of intestinal barrier.

### YAP is required for resistance to *P*. *aeruginosa* PA14 or *S*. Typhimurium in mice

**Fig 8:**
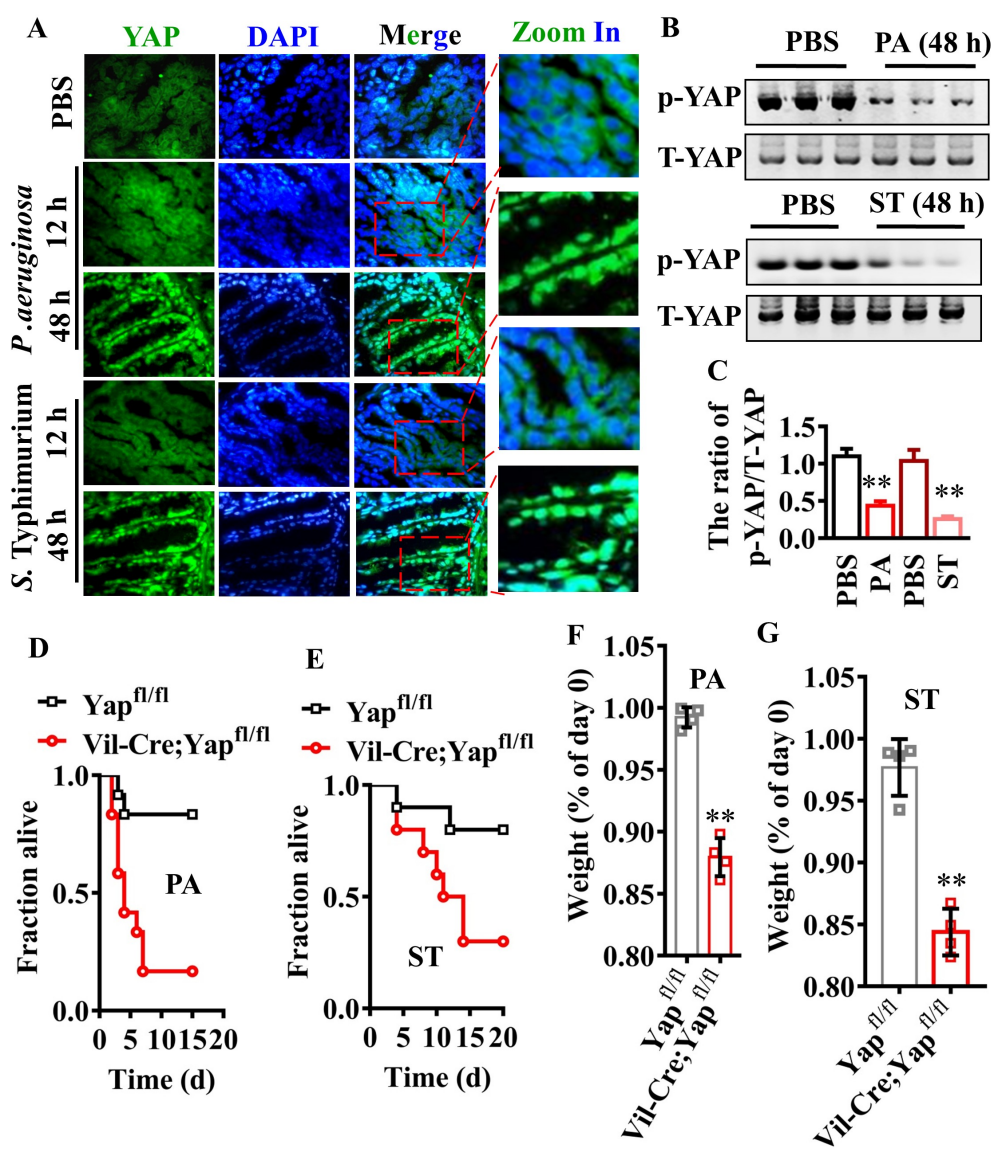
YAP is required for resistance to bacterial infection in mice. **(A)** *P*. *aeruginosa* (PA) or *S*. Typhimurium (ST) infection promoted the activation of YAP in the colon of mice after 48 h of infection. The nuclear translocation of YAP was detected by immunofluorescence staining. The parts on the right side are high-power images. **(B)** The phosphorylated levels of YAP detected by western blotting. The blot is typical of three independent experiments. **(C)** Quantification of the ratio of phosphorylated YAP-1 (p-YAP-1) to total YAP (T-YAP-1). These results are means ± SD of three experiments. **P < 0.01 relative to control (PBS). **(D and E)** Knockout of Yap in the epithelium of the small intestine and colon reduced survival rate after *P*. *aeruginosa* **(D)** or *S*. Typhimurium **(E)** infection (n = 15 each group). *P* < 0.05 relative to Yap^fl/fl^ (Log-rank test). **(F and G)** Knockout of Yap in the epithelium of the small intestine and colon accelerated the loss of body weight after *P*. *aeruginosa* **(F)** or *S*. Typhimurium **(G)** infection These results are mean ± SD of four independent experiments (n = 8/condition). **P < 0.01 relative to Yap^fl/fl^. *p*-Values (C, F and G) were calculated using two-sample t-test.

To test whether a similar phenomenon exists in the gut of mice, we first analyzed intestinal permeability in mice given *P*. *aeruginosa* PA14 or *S*. Typhimurium by gavage. Both pathogens dramatically induced intestinal barrier permeability, as measured by the dissemination of FITC-dextran 40 kD from the intestine into the bloodstream at 48 hours, but not 12 hours, after infection (S8A Fig). To evaluate the protein levels of E-cadherin, we performed immunofluorescence staining of the intestinal tissues with antibodies against E-cadherin. Immunofluorescence staining revealed that the protein levels of E-cadherin in the colon of mice were markedly decreased at 48 hours, but not 12 hours, after *P*. *aeruginosa* or *S*. Typhimurium infection (S8B Fig). Immunofluorescence staining also showed a significant increase in the nuclear translocation of YAP in the colon of mice infected with *P*. *aeruginosa* PA14 or *S*. Typhimurium at 48 hours (Fig 8A). Meanwhile, the levels of phosphorylated YAP in the colon were markedly decreased 48 hours after *P*. *aeruginosa* PA14 or *S*. Typhimurium infection (Fig 8B and 8C). To test the role of YAP in host defense against bacterial infections in vivo, we knocked out the allele of Yap in the epithelium of the small intestine and colon by crossing floxed YAP mice with Villin-Cre (Vil-Cre) mice. Furthermore, we found that Vil-Cre;Yap^fl/fl^ mice showed a significant reduction in survival rate (Fig 8D and 8E) and body weight (Fig 8F and 8G) after *P*. *aeruginosa* PA14 or *S*. Typhimurium infection. These results indicate that YAP is sufficient to confer protection against bacterial pathogenesis in mice.

## Discussion

In the *Drosophila*, Yorkie is activated in response to intestinal epithelial cell damage, and mediates a nonautonomous increase in proliferation and differentiation of intestine stem cells, thereby promoting intestinal regeneration [15–18]. In this study, we characterized a mechanism by which the intestinal epithelium detects danger and triggers innate immune responses to bacterial infection during a breach of the intestinal epithelial barrier. The E-cadherin-catenin complex, the functional unit of AJs, interacts with YAP-1/YAP in the intestine of worms under normal conditions. When the intestinal barrier is infected, disturbances of the HMR-1/E-cadherin, HMP-1/α-catenin, and HMP-2/β-catenin result in the loss of inhibition of YAP-1/YAP in a PP2A dependent manner. Upon activation, YAP-1/YAP is translocated into the nucleus, where it activates EGL-44/TEAD-mediated transcription. Both *yap-1* and *egl-44* are required for resistance to bacterial infection in worms and mice. Importantly, conservation of such delicate danger-detecting machinery embedded within the adhesion architecture may represent an ancient immune surveillance program in the intestinal epithelium of multi-cellular organisms.

The YAP-TEAD complex is widely involved in cell proliferation, differentiation, organ size, tissue regeneration, and stem cell self-renewal [42, 43]. Recent studies indicate that YAP plays sophisticated roles in innate immune responses to different types of pathogens [27, 28, 31, 32]. YAP is involved in innate immune responses to pathogen infections mainly by influencing multiple immune signaling pathways. Our work here demonstrates that the complex of YAP-1/YAP and EGL-44/TEAD are required for resistance to bacterial infection in worms and mice. The complex function as a transcription factor to regulate the expression of immune response-related genes in worms during bacterial infection. In human umbilical vein endothelial cells, overexpression of YAP induces expression of a set of pro-inflammatory genes, such as IL-6, VCAM1, and ICAM1 [45]. Likewise, hepatocyte-specific overexpression of YAP in mice upregulates the expression of inflammatory factors, including IL-1β and TNFα [46]. Thus, YAP may directly regulate a set of genes related to immune and inflammatory responses. The PMK-1-ATF-7 signaling plays a crucial role in innate immunity in *C*. *elegans* [47, 48]. A recent study has demonstrated that the PMK-1-ATF-7 signaling causes broad transcriptional changes including the upregulation of many immune-related genes after *P*. *aeruginosa* infection [41]. However, our results demonstrate that the expression of genes regulated by the EGL-44/TEAD-YAP-1/YAP complex are independent of the PMK-1-ATF-7 signaling (S9 Fig). Previous studies indicate that *pmk-1* mutation leads to increased accumulation of *P*. *aeruginosa* PA14 in the gut of worms at 20–24 hours of infection [47, 49]. In contrast, significant accumulation of *P*. *aeruginosa* or *S*. *Typhimurium* in *yap-1(tm1416)* and *egl-44(mt2247)* mutants only occurred at 48 hours, but not 24 hours. These data implicate that the EGL-44/TEAD-YAP-1/YAP signaling exhibits immune function only after the complex is activated during disruption of intestinal barrier at a late stage of bacterial infection. Thus, these two pathways function in parallel to regulate immune-related genes during pathogen infection.

It has been shown that YAP interacts with proteins found in TJs [23, 24] and AJs [25, 26]. AJs and the E-cadherin-catenin complex have been found to inhibit epithelial proliferation through regulating YAP activity [50, 51]. For instance, E-cadherin mediates contact inhibition of proliferation by promoting the cytoplasmic localization of YAP in a Hippo signaling-dependent manner [50]. Conversely, overexpression of YAP blocks the E-cadherin–mediated contact inhibition of proliferation. Moreover, disruption of AJs by calcium depletion, and E-cadherin or α-catenin RNAi in confluent Caco-2 cells promotes nuclear translocation of YAP [51]. Our data showed that disturbance of E-cadherin, α-catenin, or β-catenin by bacterial infections led to the nuclear translocation and activation of YAP. Our discoveries regarding the function of the YAP-TEAD pathway provide additional insights into the role of the E-cadherin-catenin complex in immunity and inflammation. In mammals, the AMOT family proteins of TJs also regulate YAP activity by recruiting YAP to TJs, thus inhibiting its nuclear translocation [23, 24]. However, we found that genetic ablation of several components of TJs failed to induce the nuclear translocation of YAP-1/YAP in *C*. *elegans*. One possible explanation is that unlike the components of AJs, the TJ components in *C*. *elegans* are not conserved.

It has been well-established that the Hippo pathway kinases LATS1 and LATS2 mediate the degradation of YAP [22, 42]. In this study, bacterial infections reduced the phosphorylation of YAP-1/YAP in *C*. *elegans* during the disruption of intestine barrier. The fact that the phosphorylation of WST-1/LATS1 is not altered implicates that the Hippo signaling itself is unlikely to be involved in the dephosphorylation of YAP-1/YAP during loss of the epithelial barrier. A previous study has demonstrated that α-catenin binding prevents YAP dephosphorylation in murine epidermal cells [25]. Loss of α-catenin leads to an efficient association of YAP and PP2Ac, which potentially dephosphorylates YAP. Consistent with the observation, our data showed that knockdown of *hmr-1*/E-cadherin-mediated dephosphorylation of YAP-1/YAP was also required for PP2A in the intestine of worms. Meanwhile, PP2A is involved in the activation of YAP-1/YAP in worms after bacterial infections. Thus, PP2A is a critical regulator of YAP activation during loss of epithelial barrier.

Interestingly, compromise of the intestinal epithelial barrier occurs commonly in patients with inflammatory bowel disease (IBD) and celiac disease [3, 52–54]. These diseases are characterized by excessive activation of the immune and inflammatory responses. Most current studies attribute the link between intestinal barrier disruption and inflammation to the loss of the physical barrier function and subsequent permeation of luminal bacterial products and antigens and neutrophil transepithelial migration [3, 52]. Our findings suggest a central role of the epithelial cells in triggering inflammatory responses after the intestinal barrier is damaged. Thus, our results implicate that the YAP-TEAD pathway likely contributes to the pathogenesis of IBD and celiac disease that involves chronic inflammation of the digestive tract. Therefore, interventions that suppress the YAP-TEAD pathway might provide new therapeutic avenues for IBD and other inflammatory intestinal diseases associated with barrier loss.

## Materials and methods

### Ethics statement

All mouse experiments, maintenance and care were performed according to protocols approved by the Animal Care and Use Committee of Yunnan University (Permit Number: yuncare20190034). The investigation conforms to the Guide for the Care and Use of Laboratory Animals, published by the US National Institutes of Health. All mice were weighed and monitored daily to ensure animal welfare.

### Nematode strains

Mutations and transgenic strains used in this study include *egl-44(mt2247)* and strains expressing *clec-85p*::*gfp*, *hmr-1p*::*hmr-1*::*gfp*, and *hmp-1p*::*hmp-1*::*gfp*, kindly provided by the Caenorhabditis Genetics Center (CGC; http://www.cbs.umn.edu/CGC), which is funded by NIH Office of Research Infrastructure Programs (P40 OD010440). *yap-1(tm1416)* was kindly provided by the National BioResource Project at Tokyo Women's Medical University School of Medicine. The strain that expresses *yap-1p*::*yap-1*::*gfp* was kindly provided by Dr. Y Hata (Tokyo Medical and Dental University, Japan). Mutants were backcrossed three times into the N2 strain used in the laboratory. All strains were maintained on nematode growth media (NGM) and fed with *E*. *coli* OP50 at 20°C [55].

### Animals

Kunming mice were obtained from the Animal Center, Kunming Medical University, and used at the ages of 3–4 months. Yap^flox/flox^ and Villin-Cre mice were purchased from the Jackson Laboratory and National Resource Center of Model Mice (NRCMM) respectively. To generate a mouse in which Yap was conditionally deleted in the epithelium of the small intestine and colon, Yap^flox/flox^ mice were bred to Villin-Cre mice.These mice were kept under a constant 12-hour light-dark cycle, and were allowed to eat and drink ad libitum.

### Infection of worms with bacteria

Synchronized populations of worms were cultivated at 20°C until they reached maturity. For survival assays of worms, 75 μg/ml of 5’-fluoro-2’-deoxyuridine (FUdR) was added to the assay plates to abolish the growth of progeny [56]. *P*. *aeruginosa* PA14 (a gift from Dr. K Zhu, Institute of Microbiology, CAS), *S*. *enterica* Typhimurium 468, and *S*. *aureus* ATCC 25923 (a gift from Dr. WH Lee, Kunming Institute of Zoology, CAS) were cultured in Luria broth (LB), then seeded on slow-killing plates as described previously [48]. *P*. *aeruginosa* or *S*. Typhimurium was incubated first for 24 h at 37°C and then for 24 h at 25°C. 50–60 young adult animals were transferred onto the plates at 25°C. The numbers of living worms were measured at 12 h intervals. Immobile worms unresponsive to touch were scored as dead. Worms that crawled off the plate were censored. Three plates of each genotype were used per assay and all experiments were performed four times. The sizes of sample for each assay are provided in S4 Table.

### Intestinal barrier function assay in worms

Intestinal barrier function was determined according to the method described previously with some modifications [57]. Briefly, 50–60 synchronized young adult animals were transferred onto the slow-killing plates containing pathogenic bacteria at 25°C. After 6–48 h of infections, the worms were removed from the NGM plates and suspended for 2.5 h in M9 liquid medium containing *E*. *coli* OP50 (OD = 0.5–0.6), either 5% food dye FD&C Blue No. 1 (Bis[4-(N-ethyl-N-3-sulfophenylmethyl) aminophenyl]-2-sulfophenylmethylium disodium salt) (AccuStandard, New Haven, CT) or 15 μg/ml of FITC-dextran of 40 kD (Sigma, St. Louis, MO). Animals were collected and washed four times with M9 buffer. Then the worms were mounted in M9 onto microscope slides. The slides were viewed using a Zeiss Axioskop 2 Plus fluorescence microscope (Carl Zeiss, Jena, Germany) to observe the leakage of the dyes in the body cavity. The rate of body-cavity leakage was calculated as a percentage by dividing the number of worms with dye leakage by the number of total worms. For each time point, three independent experiments were carried out. In each experiment, 50–60 of worms were calculated.

### RNA-Seq analysis

After 24 h of infection by *P*. *aeruginosa* PA14, worms were collected and sent to Novogene Corporation (Beijing, China) for RNA-sequencing analysis. *P* < 0.05 and fold change > 2 was used as a threshold for differential expression. The GEO accession number is GSE 121091.

### Identification of CATTCC Motif in the Promoters of *P*. *aeruginosa*-Induced genes expressed in intestine

We extracted upstream sequences for 832 genes responsive to *P*. *aeruginosa* PA14 infection from Ensembl. Shared upstream motifs were identified by using the BioProspector program [39]. To compare the prevalence of identified motifs in promoters of the genes, we searched these promoters using MAST [58]. A motif occurrence is defined as a position in the sequence whose match to the motif has position p-value less than 0.0001. The CATTCC motif logo was prepared using WebLogo (http://weblogo.berkeley.edu).

### RNA interference

RNAi bacterial strains containing targeting genes were obtained from the Ahringer RNAi library [59]. In the current study, all clones were verified by sequencing. RNAi feeding experiments were performed on synchronized L1 larvae at 20°C. Briefly, *E*. *coli* strain HT115(DE3) expressing dsRNA was grown overnight in LB broth containing 100 μg/ml ampicillin at 37°C, then spread onto NGM plates containing 100 μg/ml ampicillin and 5 mM isopropyl 1-thio-β-D-galactopyranoside (IPTG). The RNAi-expressing bacteria were grown overnight at 25°C. Synchronized L1 larvae were placed on RNAi plates at 20°C until they reached maturity. Young adult worms were used for further assays.

It should be noted that the following mutants exhibited lethal phenotypes: *hmr-1*, *hmp-1*, *hmp-2*, *sur-6*, *paa-1*, *let-92*, *pptr-2*, *rsa-1*, *pph-6*, *pph-4*.*1*, and *ajm-1*. We thus tested the functions of these genes by RNAi. We knockdown *hmr-1*, *hmp-1*, *hmp-2*, *paa-1*, *let-92*, *pptr-2*, *pph-6*, *pph-4*.*1*, and *ajm-1* in a 1/4 dilution, and knockdown *sur-6* and *rsa-1* in a 1/10 dilution. Knockdown of these genes in dilution results in a decrease in their expressions (S10 Fig). For double RNAi experiments, the parental worms (P0) are subjected to *hmr-1* RNAi in a 1/4 dilution. Then the offspring generations (F1) are subjected to *yap-1* and *egl-44* RNAi without dilution, *paa-1* and *let-92* RNAi in a 1/4 dilution, and *sur-6* RNAi in a 1/10 dilution.

### Construction of *hmp-1* RNAi

To generate a clone directed against *hmp-1*, a 1075 bp DNA fragment of *hmp-1* was chemically synthesized (Generay Biotech Co., Shanghai, China), and subcloned into a NotI-KpnI linearized L440 feeding vector [59]. The resulting vector containing *hmp-1* was confirmed by DNA sequencing.

### YAP-1/YAP Nuclear localization assay

After bacterial infections or treatment with RNAi, the worms expressing *yap-1p*::*yap-1*::*gfp* were immediately mounted in M9 onto microscope slides. The slides were viewed using a Zeiss Axioskop 2 Plus fluorescence microscope. The status of YAP-1/YAP localization was categorized as cytosolic localization or nuclear localization when localization was observed throughout the body from head to tail. When nuclear localization was visible, but not completely throughout the body, the status of YAP-1/YAP localization was characterized as intermediate localization. The numbers of worms with each category of YAP-1/YAP localization were counted. About 100 nematodes were counted in each experiment.

### Infection of Mice with *P*. *aeruginosa* or *S*. Typhimurium

To induce infections, SPF mice were infected by gavage with the doses of 1×10^7^ CFU of *P*. *aeruginosa* or *S*. Typhimurium expressing GFP for 12 to 72 hours. Then the mice were injected by gavage with FITC-dextran 40 KD (600 mg/kg body weight; Sigma) dissolved in sterile PBS buffer 4 hours before they were anesthetized and sacrificed. Then ~0.5 ml of blood was collected by cardiac puncture for measurement of intestinal permeability. Meanwhile, the colon tissues and feces were collected and washed with PBS buffer three times. The tissues were excised for immunofluorescence staining and western blotting.

### Measurement of Intestinal Permeability in Mice

Intestine barrier integrity was determined by FITC-dextran leakage assay. 10 μl of serum was transferred into 96-well plates in a Spectra Max M5 fluorescent microplate reader (Molecular Devices, Sunnyvale, CA) for quantification of FITC fluorescence with excitation at 488 nm and emission 518 nm. The levels of serum FITC-dextran were determined using a standard curve.

### Quantitative RT-PCR

Total RNA was extracted from worms with TRIzol Reagent (Invitrogen, Carlsbad, CA). Random-primed cDNAs were generated by reverse transcription of the total RNA samples with SuperScript II (Invitrogen). A quantitative real-time PCR analysis was performed using SYBR Premix-Ex TagTM (Takara, Dalian, China) on a Roche LightCycler 480 System (Roche Applied Science, Penzberg, Germany).The relative amount of each mRNA to mRNA of *ama-1* was calculated using the method described previously [60]. Primer pair sequences for qPCR amplification were designed using Primer-BLAST (http://www.ncbi.nlm.nih.gov/tools/primer-blast/) and are available upon request. The values of mRNA were normalized against the control gene *ama-1*.

### Western blotting

For detection of protein expression, worms and tissue samples of mice were homogenized in liquid nitrogen. Then the homogenate was lysed on ice for 60 minutes in RIPA buffer (Beyotime Institute of Biotechnology, Haimen, China). The lysates of total protein were loaded 30–50 μg per well and separated on a 10% SDS polyacrylamide gel. Proteins were then transferred to immobilon-PSQ transfer PVDF membrane (Millipore, Bedford, MA). The anti-phospho-YAP-1(Ser104) and anti-YAP-1 rabbit polyclonal antibodies were raised against peptides PIHTRQVpSAPNLH and KHQENSQQDKNQFSVH, respectively, which were obtained from Wuxi AppTec Corporation (Shanghai, China). anti-GFP (#M20004, mouse mAb, 1:1000 dilution; Abmart Inc. Shanghai, China), anti-phospho-Lats1 (Thr1079) (#9113, rabbit polyclonal antibody, 1:1000 dilution; Cell Signaling Technology) and anti-actin antibodies (ACTN05, mouse mAb, 1:1000 dilution; Abcam, Shanghai, China). The secondary antibodies used were HRP-conjugated anti-mouse (1:5000 dilution; Beijing TransGen Biotech Co., China) or rabbit IgG (1:5000 dilution; Abmart Inc.). An imaging system (Amersham Imager 600) was used for documentation of the western results. Protein intensity was analyzed using the ImageJ software (NIH). Uncropped images of Western blots are shown in S11 Fig and S12 Fig.

### Immunofluorescence staining

Mouse colon tissues were fixed in acetone for 30 min then frozen at optimal cutting temperature. Standard immunofluorescence staining was performed on 6 μm frozen sections. After blocking with the mixture of 10% normal goat serum overnight, the sections were incubated with primary antibodies specific for YAP (#4912, rabbit polyclonal antibodies,1:200 dilution; Cell Signaling Technology) and E-cadherin (#14472, mouse mAb, 1:50 dilution; Cell Signaling Technology) overnight at 4°C. The sections were washed three times in PBS with 0.1% Tween-20 (PBST) then incubated with FTIC-conjugated secondary antibodies (#111-095-003, goat anti-rabbit IgG, dilution 1:50, Jackson ImmunoResearch Laboratories, West Grove, PA) for 1 hour, respectively. After washing three times with PBST, the slides were stained with 1 μg/ml of 4,6-diamidino-2-phenylindole (DAPI) for 30 min to detect nuclei. Then the antiquenching agent was used to cover the slices. Images were acquired on a Zeiss Axioskop 2 Plus fluorescence microscope.

### Microscopy

For imaging fluorescence in worms, *clec-85p*::*gfp*, *hmr-1p*::*hmr-1*::*gfp*, and *hmp-1p*::*hmp-1*::*gfp* animals or worms stained by FITC-dextran were mounted in M9 onto microscope slides. The slides were viewed using a Zeiss Axioskop 2 Plus fluorescence microscope. Averages and standard errors were calculated based on more than 100 worms for each condition. The intensity of GFP was analyzed using the ImageJ software (NIH). Imaging of immunofluorescence staining in mouse tissues was carried out using a Zeiss Axioskop 2 Plus fluorescence microscope.

### Detection of bacterial accumulation in worms

Colony forming units (CFU) of *P*. *aeruginosa* or *S*. Typhimurium in the intestine of worms were determined according to the method described previously [61, 62]. For measurement of *P*. *aeruginosa* or *S*. Typhimurium CFU, synchronized young adult worms were exposed to plates seeded with *P*. *aeruginosa* expressing GFP (a gift from Dr. K Zhu) or *S*. Typhimurium expressing GFP (the vector for GFP expression is a gift from Dr. XP Qi, Kunming Institute of Zoology, CAS) for 24 to 72 h at 25°C. Then worms were transferred to NGM plates seeded with *E*. *coli* OP50 for 15 min to eliminate *P*. *aeruginosa* or *S*. Typhimurium stuck to the worm bodies. Animals were twice transferred to new NGM plates seeded with *E*. *coli* to further eliminate external *P*. *aeruginosa* or *S*. Typhimurium. Then worms were collected and soaked in M9 buffer containing 25 mM levamisole hydrochloride (Sangon Biotech Co., Shanghai, China) for 30 min at room temperature. For surface sterilization, the worms were transferred to M9 buffer containing 25 mM levamisole hydrochloride and 100 μg/ml kanamycin for 1 h at room temperature. Then worms were washed three times with M9 buffer. 50 nematodes per condition were transferred into 50 μl PBS plus 0.1% Triton and ground. The lysates were diluted by 10-fold serial dilutions in sterilized water and spread over LB agar plates. After one day of incubation at 37°C, colonies of GFP-*P*. *aeruginosa*, or GFP-*S*. Typhimurium were counted.

### Statistics

All experiments were performed at least three times. Differences in survival rates were analyzed using the log-rank test. Differences in mRNA and protein levels, the percentage of body cavity-leakage, and CFU were assessed by performing a one-way ANOVA followed by a Student-Newman-Keuls test. Differences in YAP nuclear accumulation were analyzed using two-sample t-test. Data were analyzed using GraphPad Prism 7.0.

## Supporting information

S1 Fig Disruption of intestinal barrier in worms exposed to *S*. Typhimurium. **(A)** Intestinal permeability measured by food dye FD&C Blue No. 1 (FD&C Blue) and FITC-dextran staining in worms exposed *S*. Typhimurium. **(B)** Quantification of body-cavity leakages in animals over time. These results are means ± SD of three independent experiments (n ≥ 50 worms per experiment). **P < 0.01 relative to 0 h (One-way ANOVA followed by a Student-Newman-Keuls test).
(TIF)

S2 Fig Comparison of gene expression between 138 differentially expressed genes containing the TEAD-binding at 24 hours and transcriptional responses at 4 hours after *P*. *aeruginosa* PA14 infection. Cluster I: The genes regulated by other transcription factors at 4 h and the TEAD/EGL-44-YAP-1/YAP complex at 24 hours; Cluster II: The genes regulated by the TEAD/EGL-44-YAP-1/YAP complex at 24 h.
(TIF)

S3 Fig Overexpression of *yap-1* confers resistance to bacterial infections in worms. **(A and B)** Worms overexpressing *yap-1p*::*yap-1*::*gfp* were more resistant to infections with *P*. *aeruginosa* PA14 **(A)** or *S*. Typhimurium **(B)** than wild type (WT) worms. *P* < 0.01 relative to WT (Log-rank test). **(C)** The colony forming units (CFU) of *P*. *aeruginosa* or *S*. Typhimurium in the worms overexpressing *yap-1p*::*yap-1*::*gfp* were significantly lower than those in WT worms. These results are mean ± SD of seven independent experiments (n ≥ 50 worms per experiment). *P < 0.05 relative to WT (Two-sample t-test).
(TIF)

S4 Fig Expression of *clec-85p*::*gfp* is upregulated in *hmr-1* RNAi-treated worms fed *E*. *coli* OP50. **(A)** Knockdown of *egl-44* and *yap-1* by RNAi reduced such an increase of *clec-85p*::*gfp* expression in *hmr-1* RNAi-treated worms. **(B)** Quantification of GFP levels. These results are means ± SD of three independent experiments (n ≥ 50 worms per experiment). **P < 0.01 (One-way ANOVA followed by a Student-Newman- Keuls test).
(TIF)

S5 Fig Bacterial infection disturbs the distribution of HMR-1::GFP and HMP-1::GFP. **(A and B)** The distribution of HMR-1::GFP or HMP-1::GFP was distributed by *P*. *aeruginosa* PA14 **(A)** or *S*. Typhimurium **(B)**. Arrows point to the HMR-1 and HMP-1 localization.
(TIF)

S6 Fig The phosphorylation levels of WTS-1/LATS1/2 are not altered after disruption of intestinal barrier. **(A)** *P*. *aeruginosa* infection did not influence the phosphorylation levels of WTS-1, which was detected using anti-phosho-Lats1 antibodies. The blot is typical of three independent experiments. **(B)** The phosphorylation levels of WTS-1 were not altered in worms subjected to *hmr-1*, *hmp-1*, and *hmp-2* RNAi under normal growth conditions (grown on *E*. *coli* OP50). The blot is typical of three independent experiments.
(TIF)

S7 Fig PP2A is required for immune responses to *P*. *aeruginosa* infection. **(A)** Unsupervised hierarchical clustering of expression levels (qRT-PCR) of immune-related genes in worms subjected to *let-92*, *paa-1*, or *sur-6* RNAi after *P*. *aeruginosa* PA14 infection by using Origin 2019b. Each column represents an independent replicate. **(B)** Knockdown of *let-92*, *paa-1*, or *sur-6* by RNAi markedly accelerated worm death. *P* < 0.01 relative to empty vector (EV) (Log-rank test). **(C)** Knockdown of *let-92*, *paa-1*, or *sur-6* by RNAi increased the colony forming units (CFU) of *P*. *aeruginosa* PA14 in worms. These results are mean ± SD of eight independent experiments (n ≥ 50 worms per experiment). **P < 0.01 relative to EV (Two-sample t-test).
(TIF)

S8 Fig Intestinal permeability is increased accompanied with a decrease in E-cadherin protein levels in the colon of mice after bacterial infection. **(A)** Intestinal permeability was measured by FITC-dextran in the serum after *P*. *aeruginosa* or *S*. Typhimurium infection (n = 8). **P < 0.01 relative to control (PBS) (Two-sample t-test). **(B)** Immunofluorescence staining revealed that the protein levels of E-cadherin in the colon of mice were markedly decreased at 48 h after *P*. *aeruginosa* or *S*. Typhimurium infection.
(TIF)

S9 Fig Venn diagram of genes that are dependent upon TEAD/EGL-44, PMK-1 or ATF-7 after *P*. *aeruginosa* infection. **(A)** Upregulated genes; **(B)** Downregulated genes.
(TIF)

S10 Fig Knockdown efficiency of RNAi. Knockdown of *hmr-1*, *hmp-1*, *hmp-2*, *paa-1*, *let-92*, *pptr-2*, *pph-6*, *pph-4*.*1*, and *ajm-1* in a 1/4 dilution, and *sur-6* and *rsa-1* in a 1/10 dilution resulted in a decrease in their expressions.
(TIF)

S11 Fig Full scans for Figs 3D, 3E, 5C and 6C. (TIF)

S12 Fig Full scans for Figs 7C and 8B, S6A and S6B. (TIF)

S1 Table Genes are upregulated in worms at 24 h after *P*. *aeruginosa* infection. (XLSX)

S2 Table Genes are downregulated in worms at 24 h after *P*. *aeruginosa* infection. (XLSX)

S3 Table The 138 *P*. *aeruginosa*-regulated genes with CATCC DNA motifs in their promoters identified by Motif Enrichment Analysis. (XLSX)

S4 Table The sizes of sample for each assay. (XLSX)
