# Supplementary figures and images for: YAP in epithelium senses gut barrier loss to deploy defenses against pathogens
Source: PLoS Pathog. 2020 Aug 28;16(8):e1008766. doi: 10.1371/journal.ppat.1008766 (PMC7454999; doi:10.1371/journal.ppat.1008766)

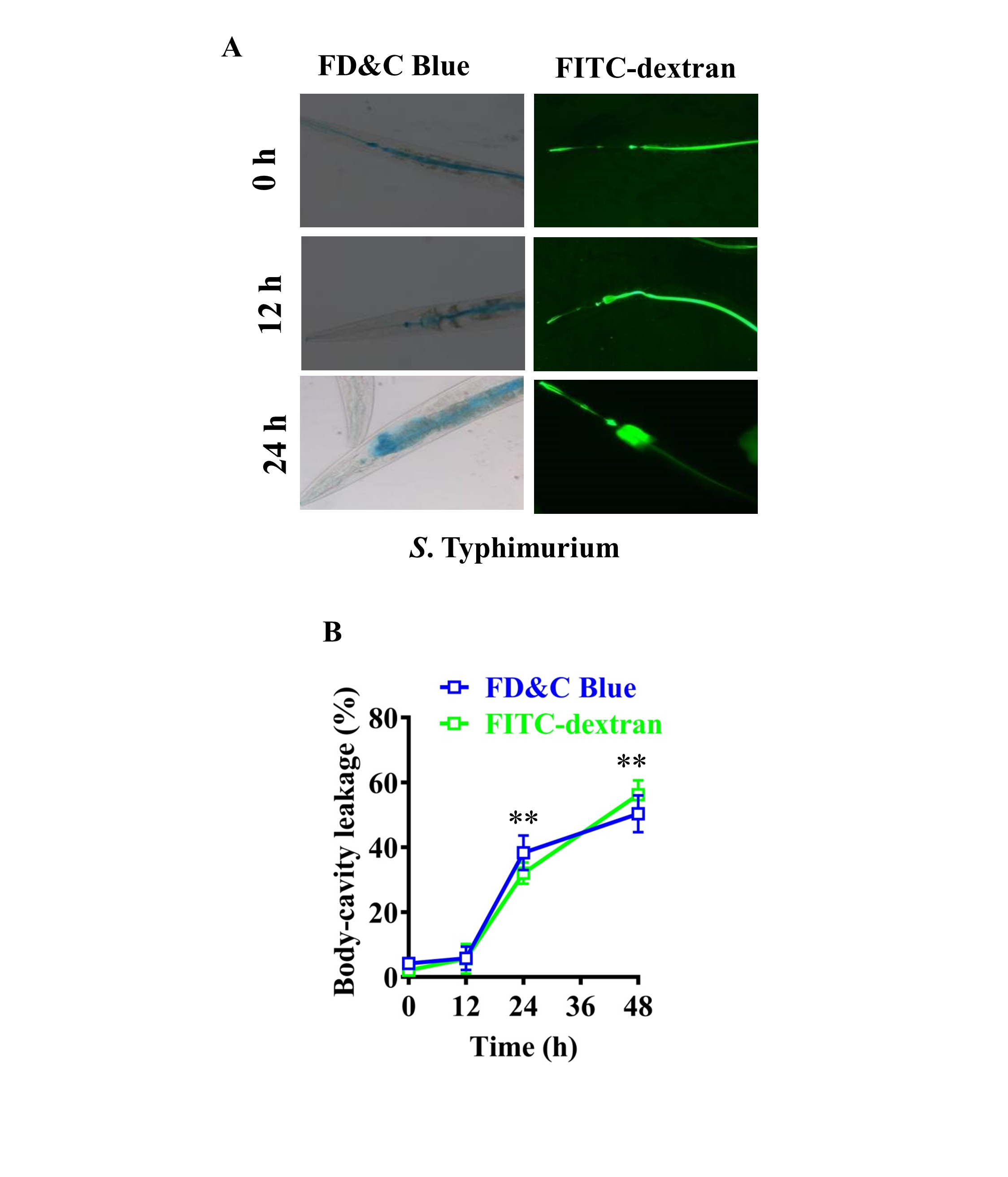

Supplement: S1 Fig — (A) Intestinal permeability measured by food dye FD&C Blue No. 1 (FD&C Blue) and FITC-dextran staining in worms exposed S. Typhimurium. (B) Quantification of body-cavity leakages in animals over time. These results are means ± SD of three independent experiments (n ≥ 50 worms per experiment). **P < 0.01 relative to 0 h (One-way ANOVA followed by a Student-Newman-Keuls test). (TIF) [file ppat.1008766.s001.TIF]

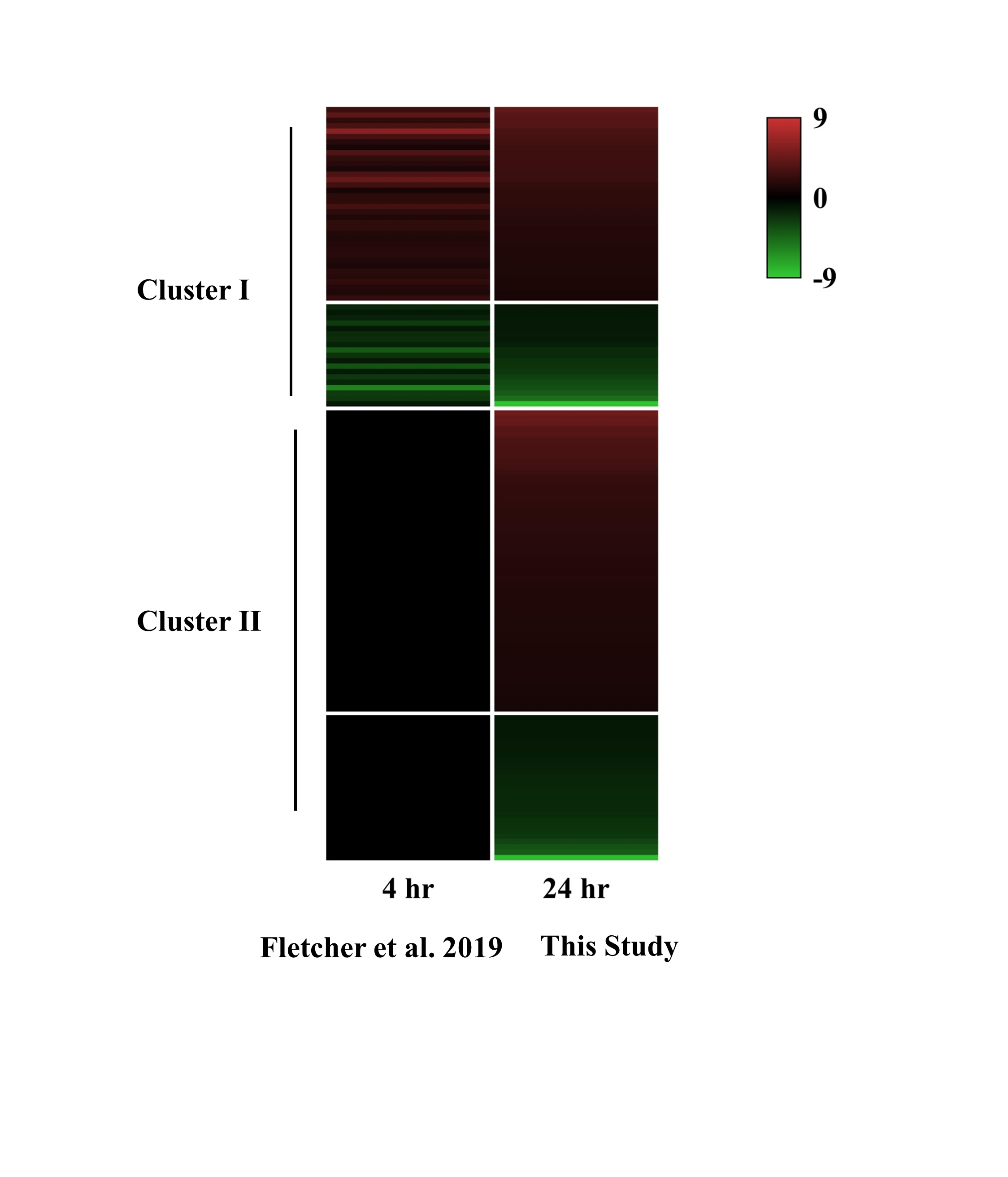

Supplement: S2 Fig — Cluster I: The genes regulated by other transcription factors at 4 h and the TEAD/EGL-44-YAP-1/YAP complex at 24 hours; Cluster II: The genes regulated by the TEAD/EGL-44-YAP-1/YAP complex at 24 h. (TIF) [file ppat.1008766.s002.TIF]

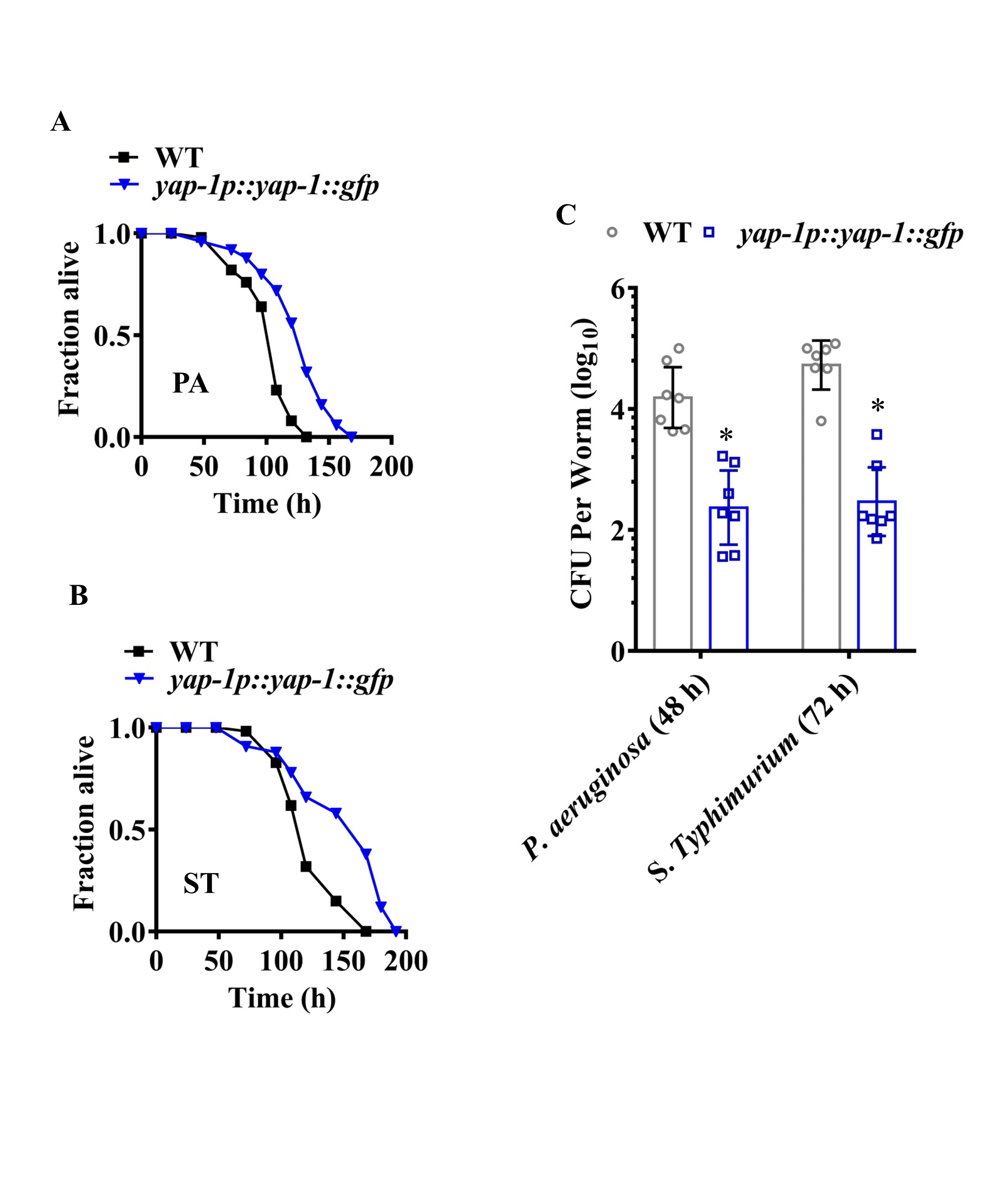

Supplement: S3 Fig — (A and B) Worms overexpressing yap-1p::yap-1::gfp were more resistant to infections with P. aeruginosa PA14 (A) or S. Typhimurium (B) than wild type (WT) worms. P < 0.01 relative to WT (Log-rank test). (C) The colony forming units (CFU) of P. aeruginosa or S. Typhimurium in the worms overexpressing yap-1p::yap-1::gfp were significantly lower than those in WT worms. These results are mean ± SD of seven independent experiments (n ≥ 50 worms per experiment). *P < 0.05 relative to WT (Two-sample t-test). (TIF) [file ppat.1008766.s003.TIF]

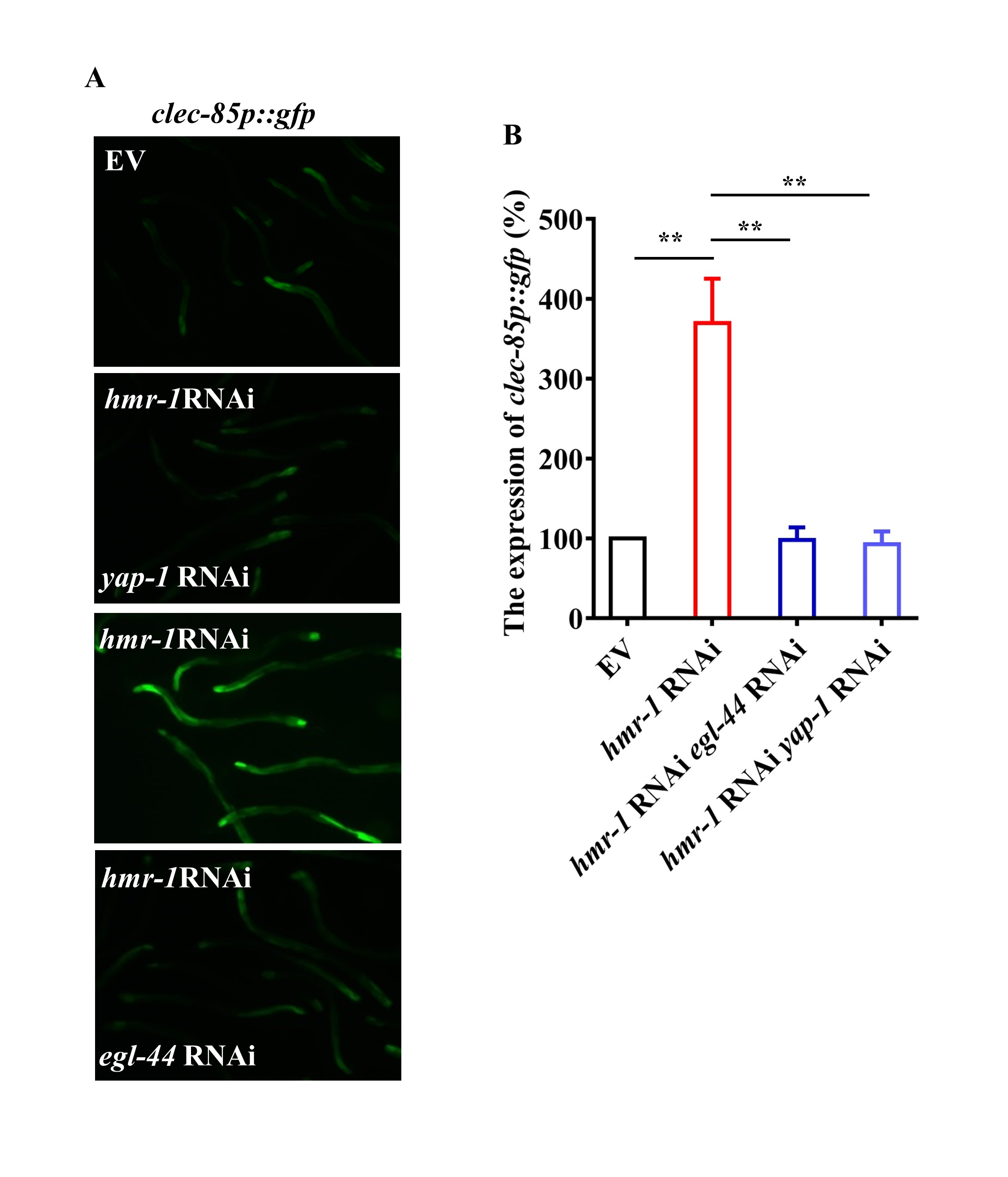

Supplement: S4 Fig — (A) Knockdown of egl-44 and yap-1 by RNAi reduced such an increase of clec-85p::gfp expression in hmr-1 RNAi-treated worms. (B) Quantification of GFP levels. These results are means ± SD of three independent experiments (n ≥ 50 worms per experiment). **P < 0.01 (One-way ANOVA followed by a Student-Newman- Keuls test). (TIF) [file ppat.1008766.s004.TIF]

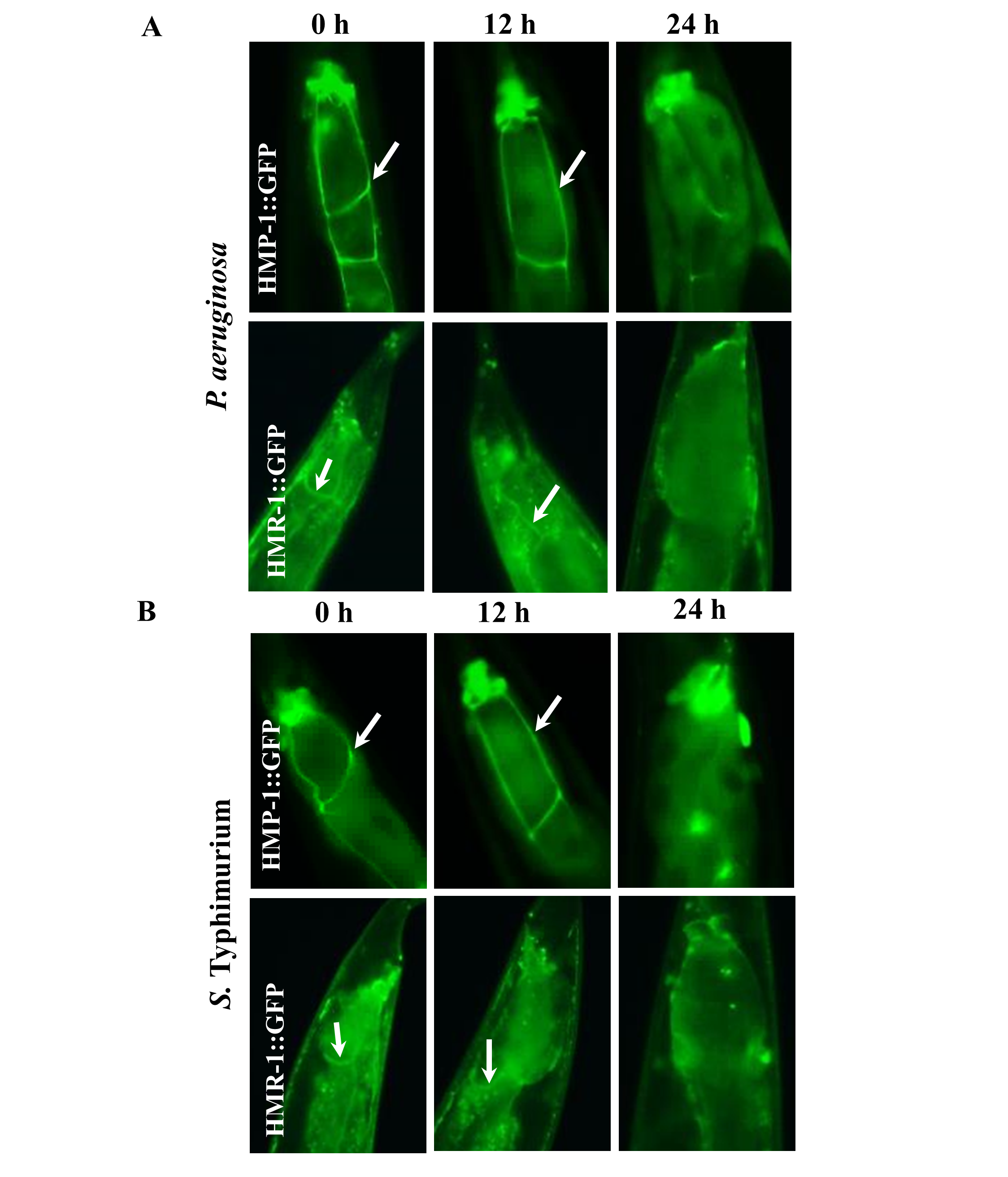

Supplement: S5 Fig — (A and B) The distribution of HMR-1::GFP or HMP-1::GFP was distributed by P. aeruginosa PA14 (A) or S. Typhimurium (B). Arrows point to the HMR-1 and HMP-1 localization. (TIF) [file ppat.1008766.s005.TIF]

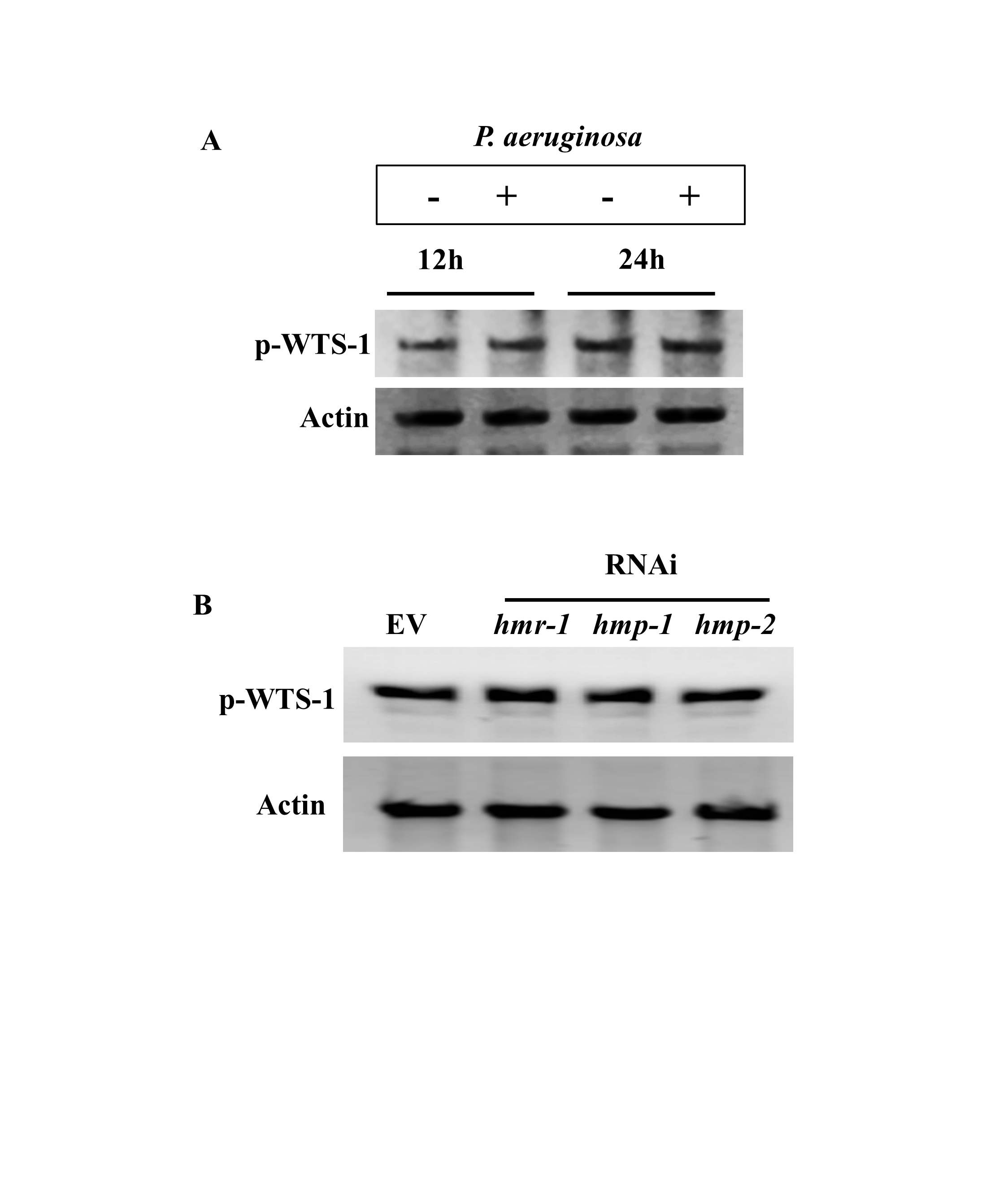

Supplement: S6 Fig — (A) P. aeruginosa infection did not influence the phosphorylation levels of WTS-1, which was detected using anti-phosho-Lats1 antibodies. The blot is typical of three independent experiments. (B) The phosphorylation levels of WTS-1 were not altered in worms subjected to hmr-1, hmp-1, and hmp-2 RNAi under normal growth conditions (grown on E. coli OP50). The blot is typical of three independent experiments. (TIF) [file ppat.1008766.s006.TIF]

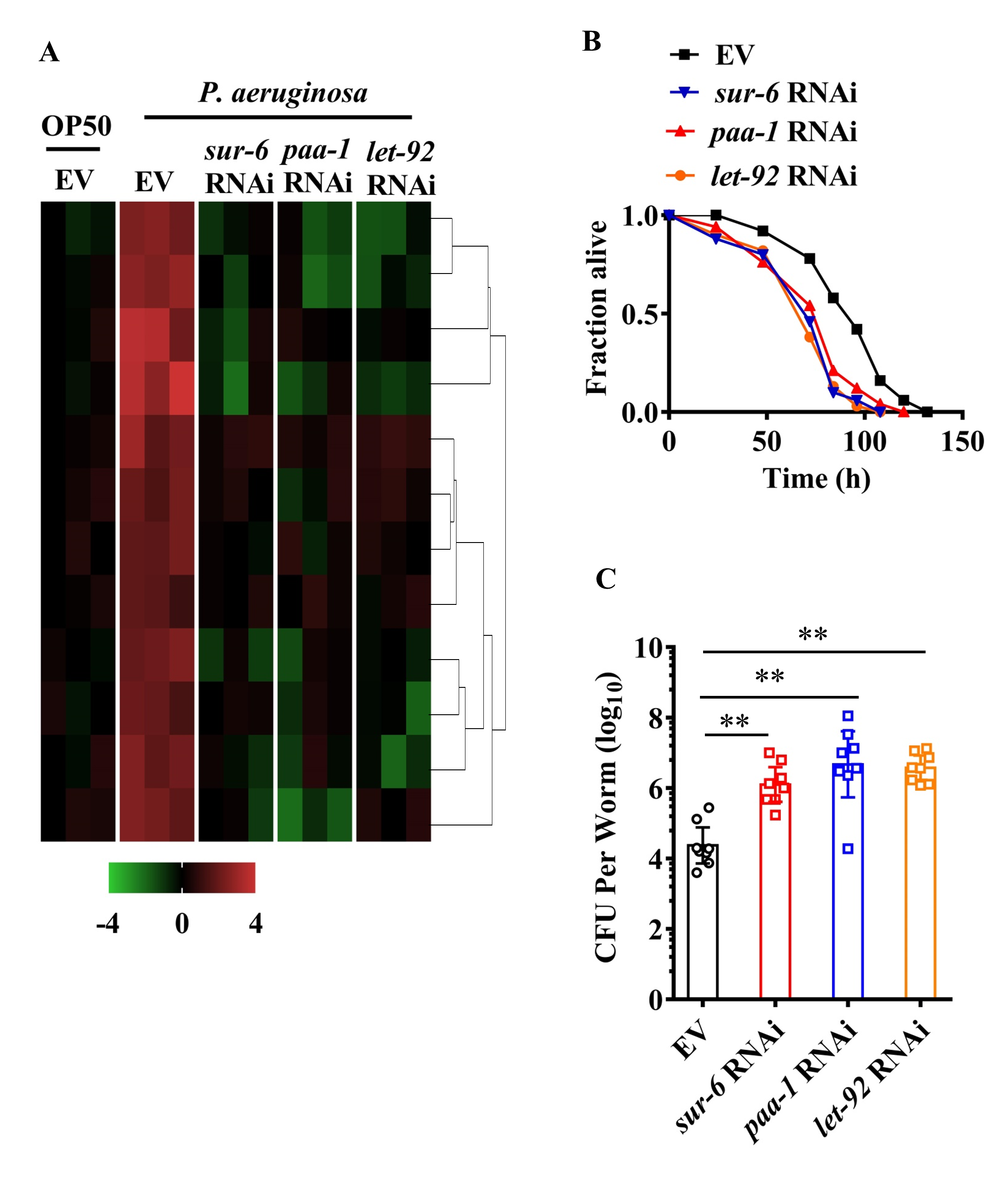

Supplement: S7 Fig — (A) Unsupervised hierarchical clustering of expression levels (qRT-PCR) of immune-related genes in worms subjected to let-92, paa-1, or sur-6 RNAi after P. aeruginosa PA14 infection by using Origin 2019b. Each column represents an independent replicate. (B) Knockdown of let-92, paa-1, or sur-6 by RNAi markedly accelerated worm death. P < 0.01 relative to empty vector (EV) (Log-rank test). (C) Knockdown of let-92, paa-1, or sur-6 by RNAi increased the colony forming units (CFU) of P. aeruginosa PA14 in worms. These results are mean ± SD of eight independent experiments (n ≥ 50 worms per experiment). **P < 0.01 relative to EV (Two-sample t-test). (TIF) [file ppat.1008766.s007.TIF]

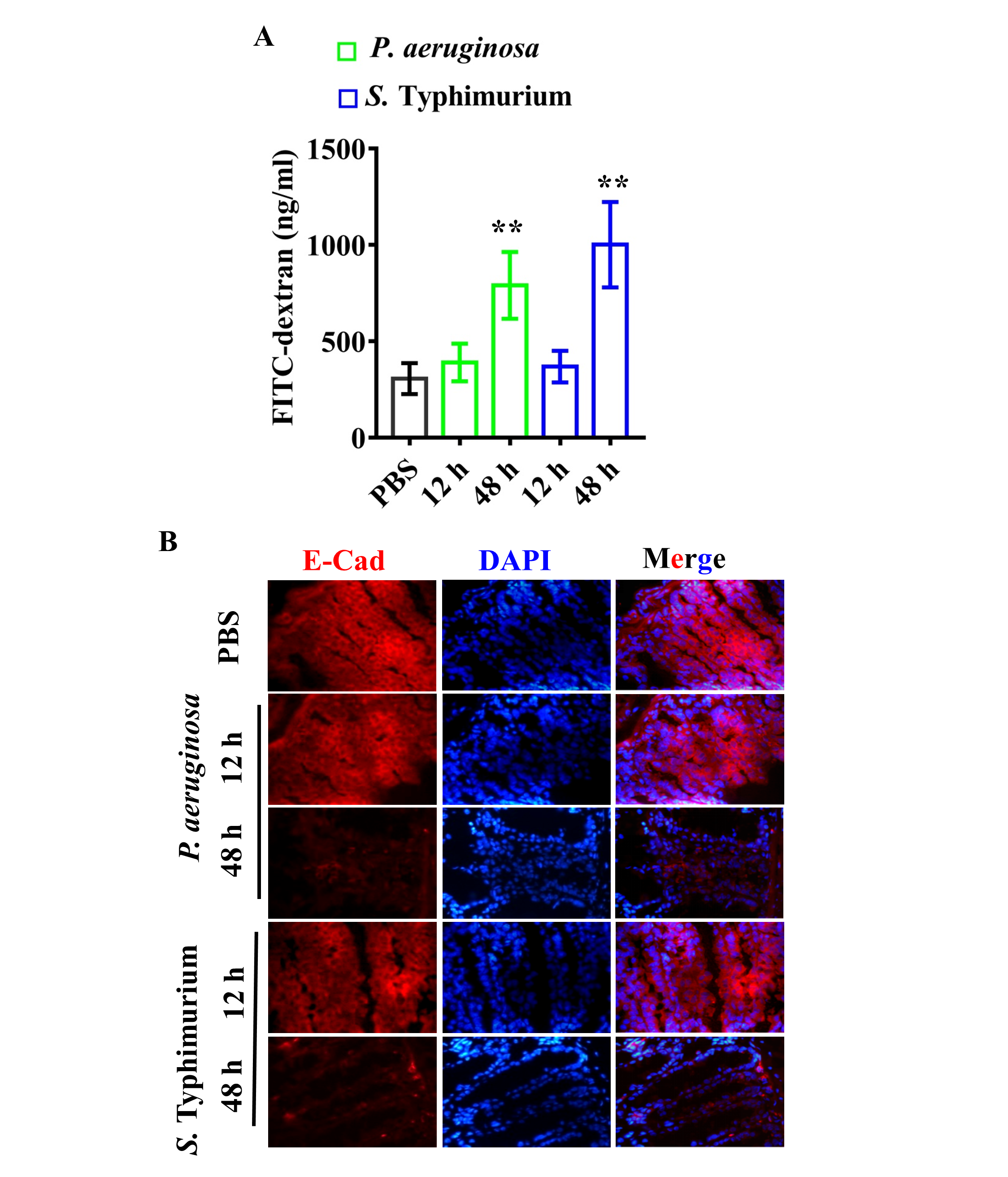

Supplement: S8 Fig — (A) Intestinal permeability was measured by FITC-dextran in the serum after P. aeruginosa or S. Typhimurium infection (n = 8). **P < 0.01 relative to control (PBS) (Two-sample t-test). (B) Immunofluorescence staining revealed that the protein levels of E-cadherin in the colon of mice were markedly decreased at 48 h after P. aeruginosa or S. Typhimurium infection. (TIF) [file ppat.1008766.s008.TIF]

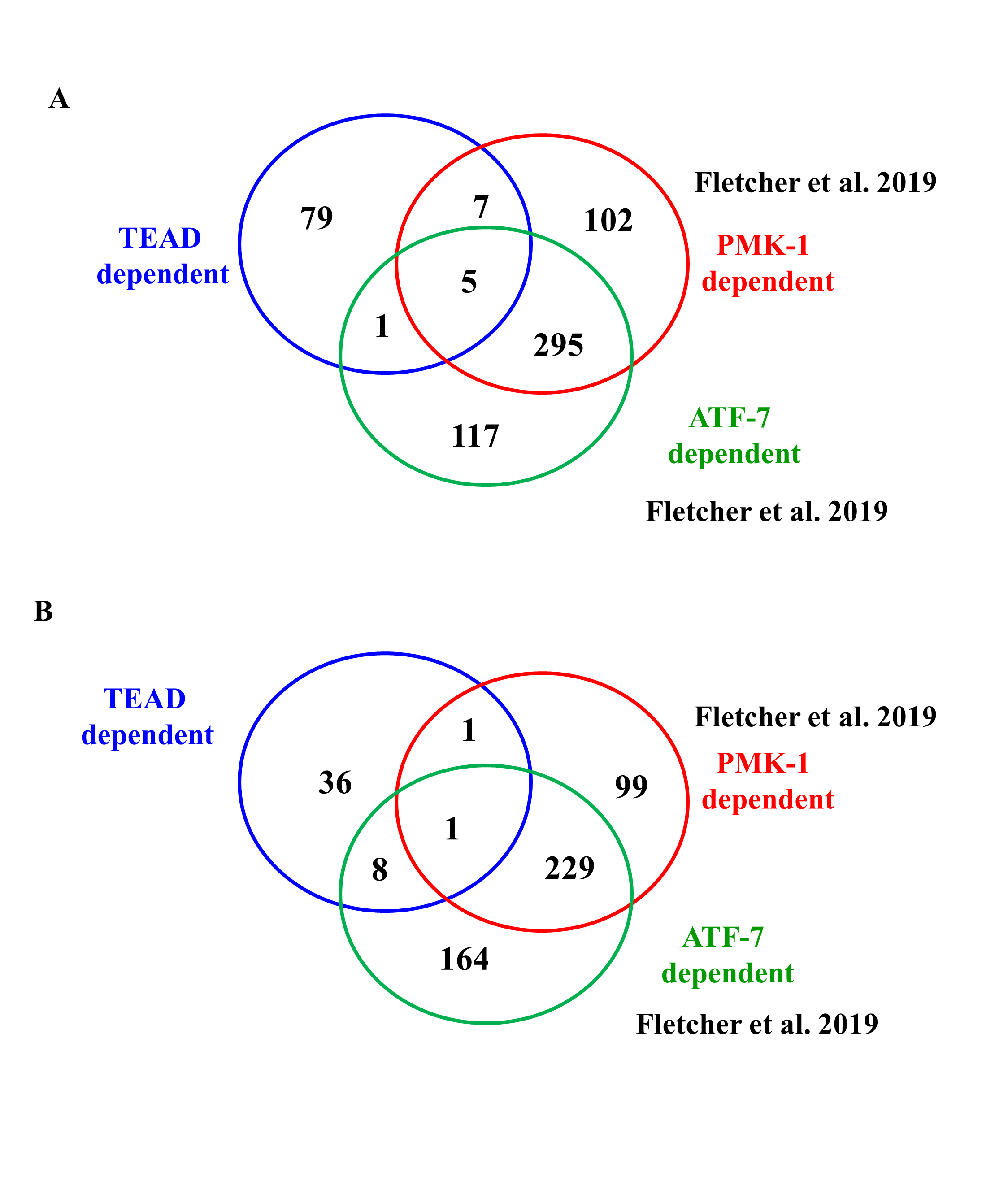

Supplement: S9 Fig — (A) Upregulated genes; (B) Downregulated genes. (TIF) [file ppat.1008766.s009.TIF]

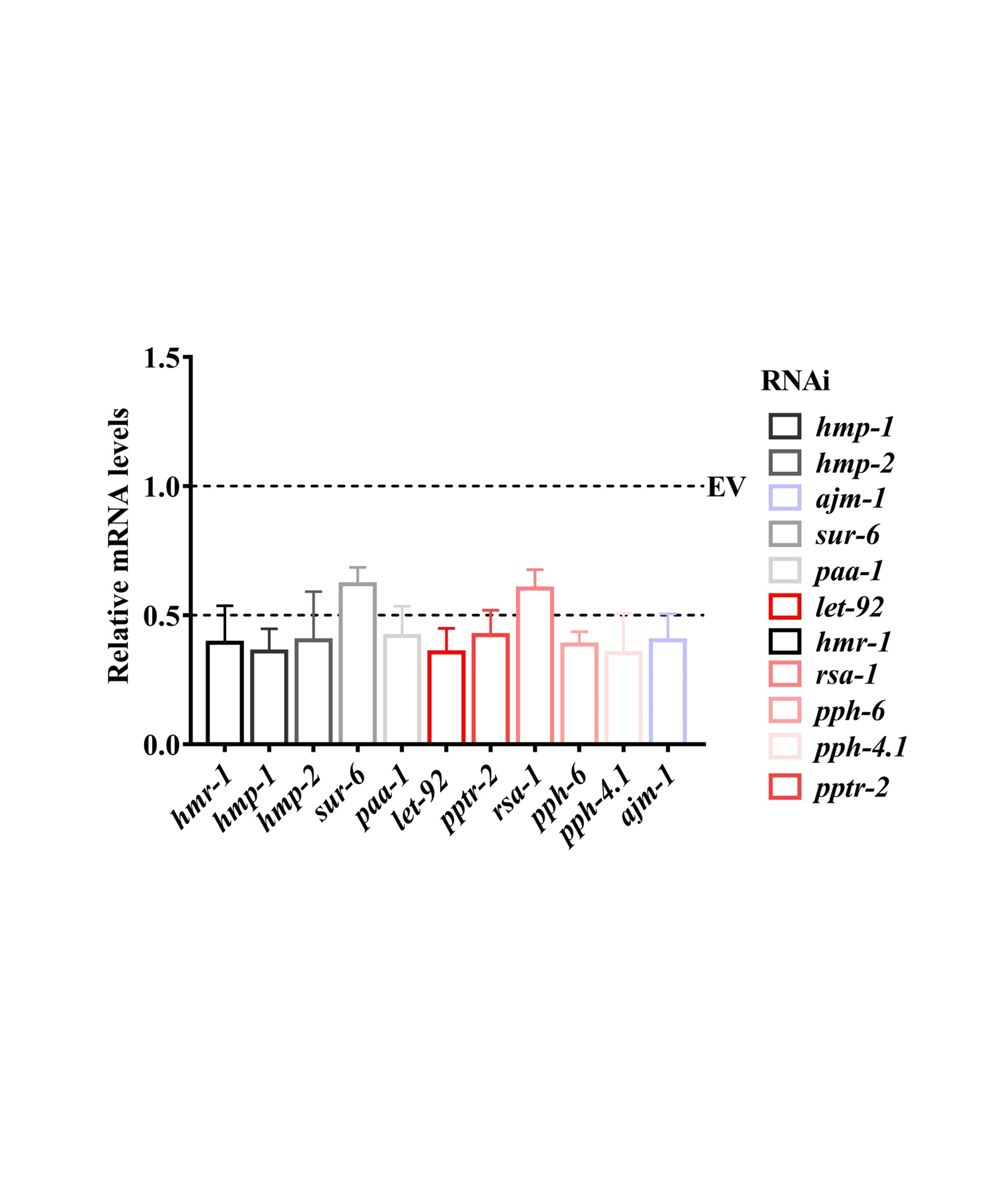

Supplement: S10 Fig — Knockdown of hmr-1, hmp-1, hmp-2, paa-1, let-92, pptr-2, pph-6, pph-4.1, and ajm-1 in a 1/4 dilution, and sur-6 and rsa-1 in a 1/10 dilution resulted in a decrease in their expressions. (TIF) [file ppat.1008766.s010.TIF]

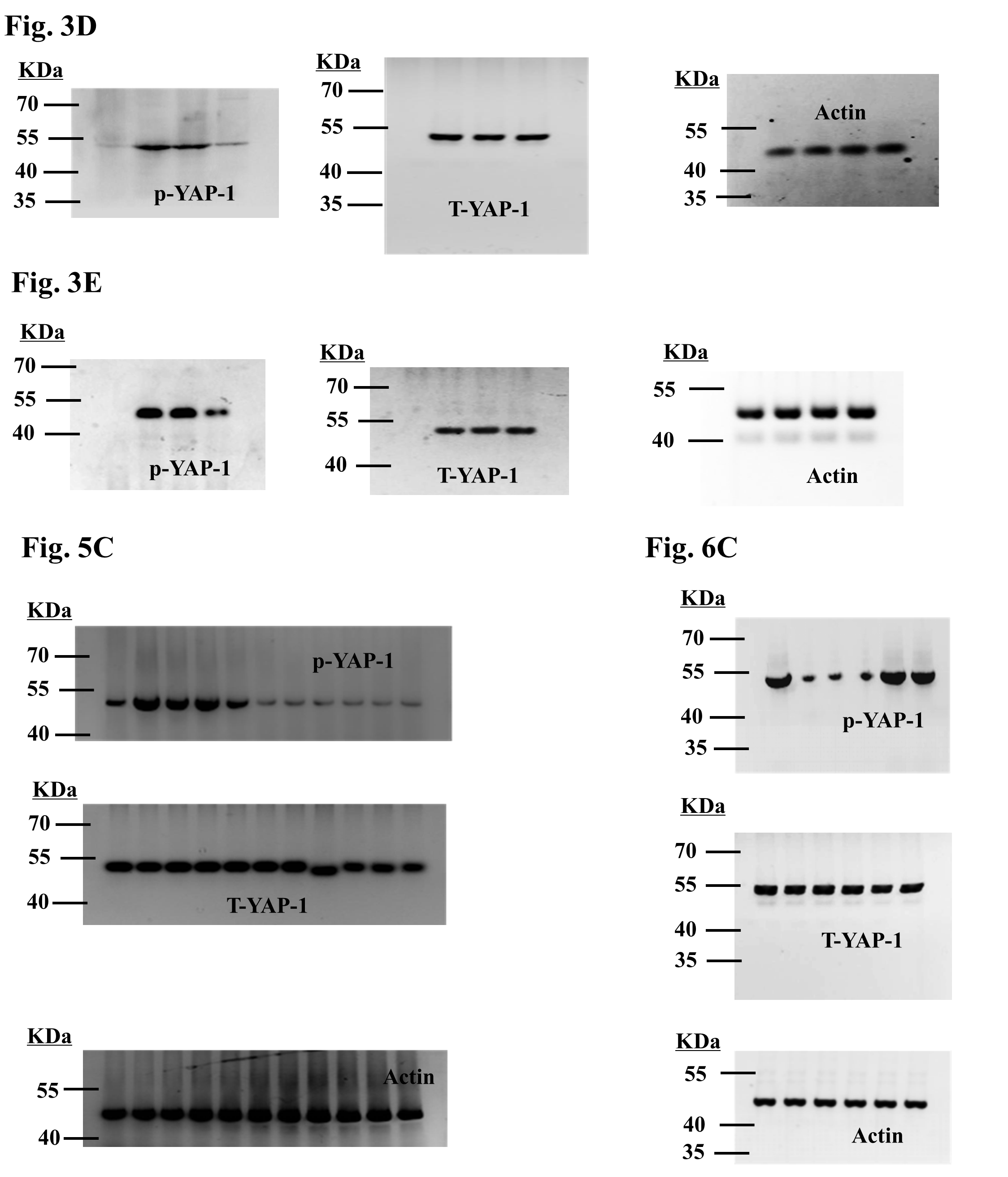

Supplement: S11 Fig — (TIF) [file ppat.1008766.s011.TIF]

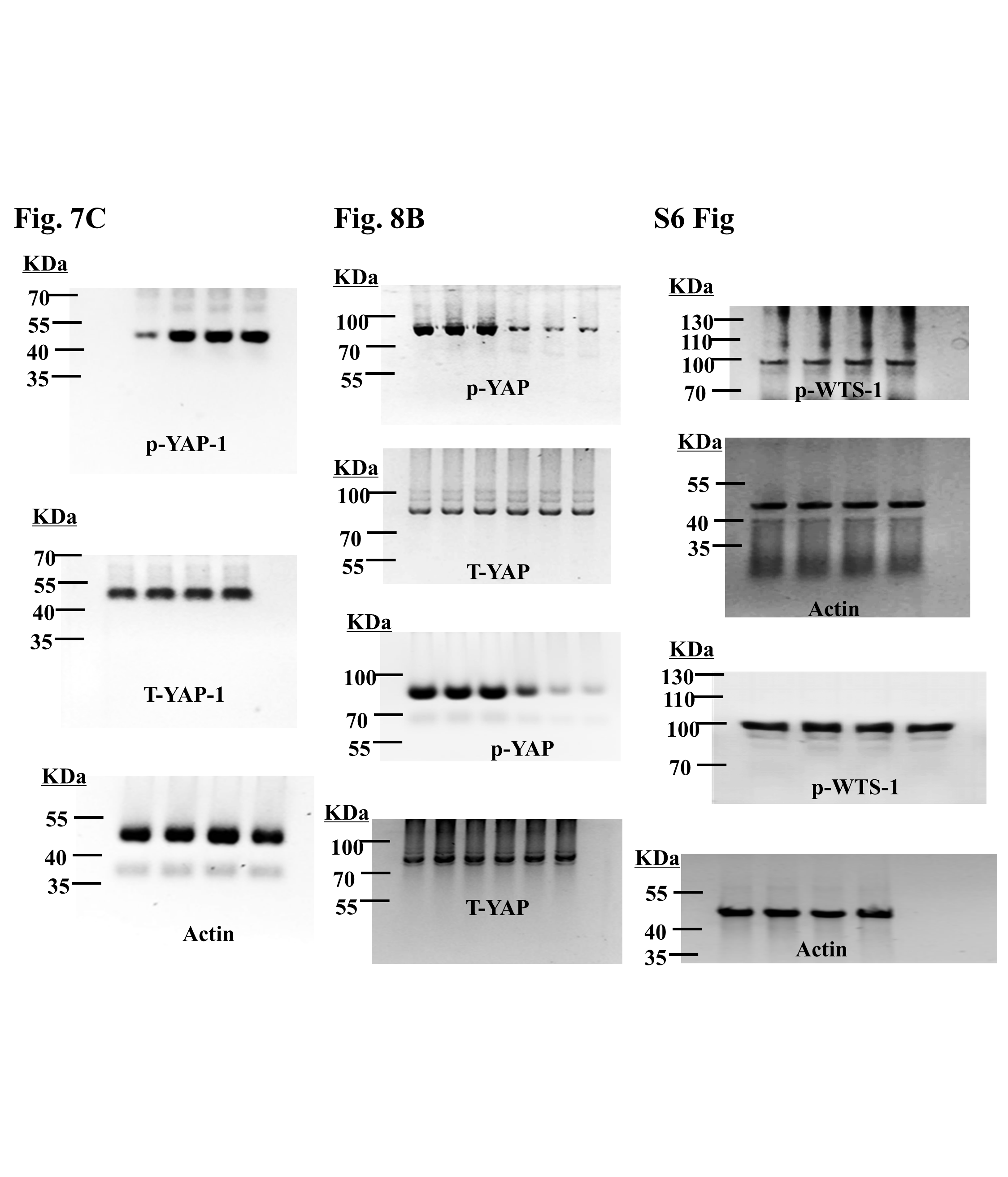

Supplement: S12 Fig — (TIF) [file ppat.1008766.s012.TIF]
